# Disentangling Shared and Differential Genetic Architectures Between COVID-19 and Other Respiratory Disorders—A Genome-Wide Multi-Omics Framework

**DOI:** 10.3390/ijms27146536

**Published:** 2026-07-22

**Authors:** Xiao Xue, Yu-Ping Lin, Yaning Feng, Hon-Cheong So

**Affiliations:** 1 School of Biomedical Sciences, The Chinese University of Hong Kong, Hong Kong SAR, China; xxxue96@gmail.com (X.X.); yupinglin50@gmail.com (Y.-P.L.); 1155149842@link.cuhk.edu.hk (Y.F.); 2 School of Medical Technology and Information Engineering, Zhejiang Chinese Medical University, Hangzhou 310053, China; 3 Department of Psychiatry, The Chinese University of Hong Kong, Hong Kong SAR, China; 4 CUHK Shenzhen Research Institute, Shenzhen 518057, China; 5 Margaret K. L. Cheung Research Centre for Management of Parkinsonism, The Chinese University of Hong Kong, Hong Kong SAR, China; 6 Brain and Mind Institute, The Chinese University of Hong Kong, Shatin, Hong Kong SAR, China; 7 Hong Kong Branch of the Chinese Academy of Sciences Center for Excellence in Animal Evolution and Genetics, The Chinese University of Hong Kong, Shatin, Hong Kong SAR, China; 8 KIZ-CUHK Joint Laboratory of Bioresources and Molecular Research of Common Diseases, Kunming Institute of Zoology and The Chinese University of Hong Kong, Hong Kong SAR, China

**Keywords:** COVID-19, respiratory disorders, multi-omics, genome-wide analysis, shared genetics, differentiation analysis, alternative splicing, interferon signaling, surfactant metabolism

## Abstract

A bidirectional relationship has been observed between COVID-19 and respiratory disorders, where respiratory comorbidities increase severity, and COVID-19 induces respiratory sequelae. The underlying biological and genetic mechanisms remain unclear. While previous studies have identified overlapping genetic loci, few have systematically disentangled the genetic factors shared between these conditions versus those specific to COVID-19, particularly at a multi-omics level. We developed and applied a unified analytical framework to compare three COVID-19 phenotypes with eight respiratory disorders (including asthma, COPD, IPF, and pneumonia). Utilizing the cofdr method for shared genetic signal analysis and DDx/mtCOJO for differentiation, we integrated genome-wide association statistics with multi-omics data (transcriptome, splicing, and proteome). This approach allowed for the simultaneous identification of shared genetic signals (concordant or discordant) and disease-specific variants across expression (TWAS), alternative splicing (spTWAS), and protein abundance (PWAS). We delineated a comprehensive atlas of 214 differential and numerous shared loci across 24 pairwise comparisons. The shared genetic architecture was characterized by pleiotropic effects in genes such as *ATP11A* (exhibiting opposing effects in COVID-19 vs. IPF) and *GSDMB* (shared with COPD). Crucially, differentiation analysis revealed that severe COVID-19 is genetically distinct from other respiratory infections (e.g., pneumonia and influenza) through dysregulated Type I/III interferon signaling and specific defects in alveolar epithelial and macrophage function, as well as GM-CSF/surfactant metabolism pathways. These findings provide human genetic evidence consistent with the therapeutic rationale underlying GM-CSF modulators and interferon-lambda for COVID-19, both of which have entered clinical trials. Furthermore, multi-trait conditional analysis prioritized *FYCO1* and *HCN3* as potential COVID-19-specific risk genes. Splicing analysis underscored the critical role of alternative splicing in both shared and differential architectures, highlighting *IFNAR2* isoform regulation as a key discriminator between COVID-19 and other respiratory traits. This study provides the first genome-wide, multi-omics map revealing the shared and differential genetic landscapes of COVID-19 and other respiratory phenotypes. By uncovering specific molecular mechanisms that distinguish COVID-19 pathology, specifically involving surfactant homeostasis and interferon pathways, our findings offer novel insights for targeted drug repurposing and precision risk stratification.

## 1. Introduction

Coronavirus disease 2019 (COVID-19), caused by severe acute respiratory syndrome coronavirus type 2 (SARS-CoV-2), has greatly impacted global health, affecting hundreds of millions of individuals and causing millions of deaths worldwide. An important feature of COVID-19 is the remarkable heterogeneity in clinical outcomes. While many experience mild symptoms, others progress to severe disease. Understanding the factors that determine this heterogeneity is of paramount importance for risk stratification, early intervention, and the development of targeted therapeutics.

Epidemiological studies have consistently identified pre-existing respiratory disorders as major risk factors for adverse COVID-19 outcomes. Chronic obstructive pulmonary disease (COPD) is associated with a substantially increased risk of severe COVID-19, hospitalization, and mortality [1,2]. Similarly, patients with interstitial lung disease (ILD) and idiopathic pulmonary fibrosis (IPF) experience poor outcomes following SARS-CoV-2 infection [3,4]. Prior influenza infection (especially within one year) has also been linked to increased COVID-19 severity [5]. The relationship between asthma and COVID-19 severity is more complex, with some studies reporting increased risk of hospitalization [6], while others suggest protective effects [7,8], a discrepancy that remains incompletely understood. It is important to note that the clinical heterogeneity of COVID-19 reflects a complex interplay of genetic, epigenetic, microbiome, and environmental factors. The present study primarily addresses the host germline genetic component of this architecture, while the contributions of epigenetic remodeling, microbiome composition, and environmental exposures are acknowledged as complementary dimensions that lie beyond the scope of our GWAS-based analysis.

Beyond serving as risk factors for acute infection, respiratory comorbidities may also emerge as sequelae of COVID-19, with growing evidence that COVID-19 survivors experience long-lasting pulmonary impairment [9,10], lung fibrosis [9], and increased susceptibility to subsequent respiratory disorders including COPD or COPD exacerbation [11].

### 1.1. Importance of Understanding Shared and Differential Genetic Architecture

The bidirectional relationships between COVID-19 and other respiratory disorders raise fundamental questions about the underlying biological mechanisms. Shared genetic factors and pathophysiology with other respiratory disorders may explain why certain pulmonary conditions serve as risk factors for severe COVID-19 and/or develop as sequelae. From a clinical perspective, identifying shared genetic factors could inform risk prediction for severe infection or sequelae, enable targeted screening of high-risk individuals, and reveal common therapeutic targets for comorbid diseases.

Conversely, the observation that only a subset of patients with respiratory comorbidities develops severe COVID-19, and that only some survivors experience pulmonary sequelae, suggests the existence of disease-specific genetic factors. In addition, studies have suggested that COVID-19 is associated with higher mortalities compared to influenza [12,13,14], suggesting that unique pathological mechanisms may underlie COVID-19. Identifying COVID-19-specific variants could illuminate unique aspects of SARS-CoV-2 pathogenesis and reveal therapeutic targets that could be modulated without affecting other respiratory conditions. Understanding both the shared and differential genetic architecture between COVID-19 and other respiratory disorders is therefore of substantial scientific and clinical importance.

### 1.2. Previous Relevant Studies and Limitations

Genome-wide association studies (GWASs) have made substantial progress in identifying genetic variants associated with COVID-19 susceptibility and severity. The COVID-19 Host Genetics Initiative (HGI) has identified numerous risk loci through international collaboration [15]. Similarly, well-powered GWASs have been conducted for respiratory disorders including asthma, COPD, IPF, and pneumonia, identifying hundreds of associated loci [16,17,18,19,20]. Several studies have begun to explore genetic overlap between COVID-19 and respiratory conditions [21,22,23,24]. For example, a cross-trait meta-analysis identified shared loci between COVID-19 hospitalization and asthma, including variants within *ABO* and *ATXN2* [22]. Another colocalization analysis detected a shared signal at rs12610495 between severe COVID-19 and IPF, implicating an expression quantitative trait locus (eQTL) for *DPP9* in lung tissue [21,25]. Genetic correlation analyses have documented significant overlap between COVID-19 and various respiratory phenotypes [25,26].

Despite the progress, several important limitations are present for existing cross-trait genetic studies of COVID-19 and respiratory disorders. First, most studies have focused on identifying shared genetic effects through colocalization or genetic correlation, without systematically examining disease-specific (differentially associated) variants or those with opposing effects between conditions. This one-sided approach provides an incomplete picture of cross-trait genetic architecture. Second, existing studies have largely operated at the SNP level, with limited integration of multi-omics data to reveal functional mechanisms. While some studies have incorporated expression QTL data [27], few have simultaneously examined splicing QTL (sQTL) and protein QTL (pQTL), despite growing evidence that alternative splicing and post-transcriptional regulation play important roles in respiratory disease pathogenesis [28,29,30,31,32]. Third, most previous analyses have examined only pairwise comparisons between COVID-19 and a single respiratory trait, rather than simultaneously analyzing multiple conditions to identify variants with consistent versus heterogeneous effects across the respiratory disease spectrum. Fourth, very few studies employ a genome-wide scan approach to examine shared or differential SNPs/genes between disorders across the entire genome. Some studies focused on a restricted set of genetic variants, for example, those passing genome-wide significance in COVID-19 and tested whether they are implicated in other disorders [33]. By ignoring the broader polygenic architecture, these studies likely miss smaller-effect variants that contribute to shared or divergent genetic risk, thereby limiting the discovery of novel biological pathways. Notably, the present study is predominantly restricted to European-ancestry cohorts, given that current large-scale GWAS summary statistics are largely limited to Europeans. The implications of this limitation are discussed further in Section 3.10.

### 1.3. Study Goals and Analytical Approach

To address these gaps, we presented and applied a comprehensive framework to systematically uncover both shared and specific genetic factors underlying COVID-19 and other respiratory disorders through integration of large-scale genetic data with multi-omics resources. Our framework has several key features that distinguish it from previous approaches.

First, we integrate shared genetic signal/colocalization analysis with differentiation analysis within a unified pipeline. Methods such as cofdr, gwas-pw and hyprcoloc identify variants with shared effects across traits, while differentiation methods (DDx, CC-GWASs, mtCOJO) identify variants with heterogeneous or disease-specific effects. Crucially, rather than relying on qualitative assessments, all selected methods provide statistical significance measures through *p*-values or posterior probabilities. By applying both approaches, we can comprehensively characterize genetic variants as shared with concordant effects, shared with discordant effects, specific to one condition, or not associated with either condition.

Second, we extend cross-trait analysis beyond SNP-level associations to gene-level effects derived from multiple “omics” layers. By considering gene-level statistics from MAGMA (positional mapping), TWAS (expression), spTWAS (splicing), and PWAS (protein abundance), we identified potential shared and differential genes and then characterized whether they influence the disorders through effects on gene expression, alternative splicing, and/or protein levels.

Third, we also attempted to prioritize and validate our findings through convergent evidence from multiple independent methods, thereby reducing false discoveries that may arise from method-specific assumptions or biases.

We applied this framework to compare GWAS data on three COVID-19 phenotypes (severe/critical COVID-19 [A2], hospitalized COVID-19 [B2], and reported SARS-CoV-2 infection [C2]) with eight respiratory disorders (asthma, ILD, IPF, COPD, pneumonia, bacterial pneumonia, viral pneumonia, and influenza), encompassing 24 pairwise comparisons and three multi-trait comparisons (Table 1). Shared and differentially associated variants and genes were identified. Enriched gene sets, tissues, and cell types were also detected. Importantly, our analytic framework has been made openly available on GitHub, and we believe this will serve as a useful resource for researchers in human genetics.

**Figure 1:**
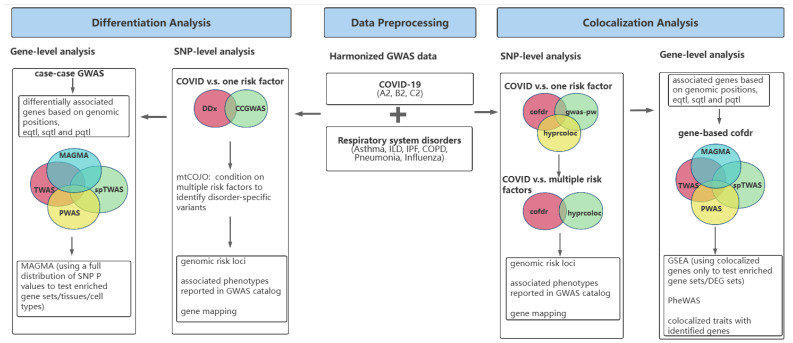
The framework of colocalization and differentiation analyses.

To our knowledge, this is the first study to employ a genome-wide and multi-omics framework to map the shared and divergent genetic landscapes between COVID-19 and a comprehensive spectrum of respiratory disorders (Figure 1). Overall, our results provide a comprehensive atlas of shared and differential genetic architecture between COVID-19 and other respiratory diseases, with implications for understanding disease mechanisms and informing personalized approaches to prevention and treatment.

## 2. Results

### 2.1. Shared Genetic Signals

#### 2.1.1. Pairwise Shared Signals at the SNP-Level

SNP-level analyses were conducted to find shared genomic risk loci between pairs of traits. In A2-related (severe/critical COVID-19) comparisons, cofdr identified 113 risk loci, of which 14 were prioritized/validated by both gwas-pw and hyprcoloc. In B2-related (hospitalized COVID-19) analyses, 119 loci were identified, with 11 validated, and in C2-related (reported COVID-19) analyses, 29 loci were identified, with five validated (Table 2). Among all validated loci, 17 had *p* < 5 × 10^−6^ in the original GWAS for both traits, with nine showing concordant allelic effects and 8 showing opposing effects (Table 3). No comparisons involving viral pneumonia yielded validated shared or differential loci, reflecting the substantially smaller effective sample size and statistical power of the viral pneumonia meta-analysis (N_cases = 2373) compared to the all-pneumonia (N_cases = 58,600) and bacterial pneumonia (N_cases = 22,399) meta-analyses, rather than a true absence of genetic overlap with COVID-19.

The comparison between A2 and idiopathic pulmonary fibrosis (IPF) yielded the highest number of validated risk loci across the 24 pairwise analyses (Table 2). The four-group cofdr model estimated that A2 and IPF share approximately 3.31% of associated SNPs. Furthermore, the likelihood ratio test provided strong evidence for a shared genetic basis between these two traits (LRT. *p* ≈ 0) (Supplementary Table S2.2).

PheWAS via GWAS Atlas revealed that five shared SNPs were significantly associated with other respiratory or immunological traits. Three SNPs (rs34517439 for A2-Asthma, rs13135092 for B2-Asthma, and rs13135092 for C2-Asthma) demonstrated consistent allelic directions, while two (rs12585036 for A2-IPF and rs75898026 for B2-IPF) exhibited opposing effects (Table 3).

#### 2.1.2. Pairwise Shared Signals at the Gene Level

By integrating GWAS data with multi-tissue transcriptome and proteome data, we identified shared genes using gene-based cofdr. We prioritized genes that were consistently identified by at least three of our four analytical pipelines: MAGMA-cofdr, TWAS-cofdr, spTWAS-cofdr, and PWAS-cofdr. This approach identified 67 validated shared genes for A2 and related respiratory disorders, 55 for B2, and 26 for C2 (Table 2). Of these, 65 genes reached FDR < 0.05 in the original MAGMA analysis for both traits. In the original TWAS analysis for lung and whole blood tissues, 23 genes were significant for both traits, with five showing concordant effect directions. Splicing-level analysis via spTWAS identified 59 genes significant in both traits, with 20 showing concordant directions. PWAS identified three shared proteins, one of which exhibited a consistent effect direction (Table 4).

The A2-IPF comparison again yielded the most validated shared genes (Table 2). Notably, TWAS-cofdr identified *ATP11A* in whole blood with a high posterior probability (π_TT = 0.97). Interestingly, the effect direction was discordant: increased expression of *ATP11A* in blood was associated with higher IPF risk but was protective against severe COVID-19 (A2). This gene was also prioritized by spTWAS-cofdr with high posterior probabilities across tissues (π_TT = 1.00 in lung; π_TT = 0.99 in blood) and by MAGMA-cofdr (π_TT = 0.99) (Table 4).

PheWAS indicated that most identified genes were associated with respiratory or immunological traits, with the exceptions of *UNC50* and *ABO*. Pathway enrichment via FUMA highlighted immunoregulatory interactions between lymphoid and non-lymphoid cells for A2-Asthma, lung function (FEV1) for A2-Asthma and B2-Asthma, and white blood cell counts for C2-COPD.

To assess robustness to the choice of posterior probability threshold, we repeated the identification of shared genes at thresholds of 0.80, 0.85, 0.90, and 0.95 (Supplementary Table S6). The number of shared genes was generally stable across thresholds of 0.80 to 0.90, with expected reductions at 0.95. Key shared genes highlighted in the main text were consistently identified across all four thresholds in the majority of relevant comparisons and omics layers, supporting the robustness of these findings. A cross-method concordance table showing which of the four omics approaches (MAGMA, TWAS, spTWAS, PWAS) identified each shared gene in each comparison is provided in Supplementary Table S7.1.

### 2.2. Multiple-Trait Shared Signals at the SNP and Gene Levels

Using a posterior probability threshold of 0.9, cofdr identified ten loci shared across A2 and four respiratory disorders (asthma, ILD, IPF, and COPD); however, these were not detected by hyprcoloc. A more permissive threshold of 0.8 yielded eight loci for B2 and two for C2 (Table 2). At the gene level, MAGMA-cofdr and TWAS-cofdr identified two and three shared genes, respectively (Supplementary Tables S3.3 and S3.4).

### 2.3. Results of Differentiation Analyses

#### 2.3.1. Pairwise Differentiation Analyses at the SNP Level

Differential association tests across the 24 pairwise comparisons were conducted using DDx and validated with CC-GWAS. We identified 69, 58, and 87 validated differentiated loci for A2, B2, and C2, respectively (Table 2). Using a significance threshold of *p* < 5 × 10^−8^, these loci were categorized into four groups: (1) 80 loci significant only in COVID-19; (2) 118 significant only in other respiratory disorders; (3) seven significant in both traits but with greater significance in COVID-19 (all showing opposing effects except rs2277732 in A2 vs. IPF); and (4) nine loci not significant for either trait individually, which could be reclassified as unique or opposing-direction loci using a more lenient threshold of *p* < 5 × 10^−6^. Particularly, PheWAS of those 80 COVID-19-unique loci revealed that 21 lead SNPs were previously associated with other respiratory or immunological studies, mostly with opposing effects.

Effect sizes for validated differential loci, as estimated by DDx, ranged from approximately |β| = 0.05 to 0.35 (on the log-OR scale). This spectrum includes both opposing allelic effects across traits and prominent COVID-19-specific signals paired with near-zero respiratory comparator effects. These DDx effect sizes reflect the case–case difference between traits rather than absolute single-disease risk. Full details, including original GWAS statistics for both traits, are provided in Supplementary Table S4.2.

#### 2.3.2. Pairwise Differentiation Analyses at the Gene Level

We merged results from four gene-level differentiation pipelines: MAGMA-DDx/CC-GWAS, TWAS-DDx/CC-GWAS, spTWAS-DDx/CC-GWAS, and PWAS-DDx/CC-GWAS. Genes identified by at least three approaches were considered validated, resulting in 95, 90, and 183 genes with differential expression/splicing/protein levels for A2, B2, and C2 comparisons, respectively (Table 2).

Among all validated genes, 99 had FDR < 0.05 in the original MAGMA gene-level analysis for COVID-19 but FDR > 0.05 for the respiratory comparator; 27 had FDR < 0.05 in the original TWAS analysis in lung and whole blood for COVID-19 but FDR > 0.05 for the comparator, all with opposing effect directions; 94 had FDR < 0.05 in the original spTWAS analysis in lung and whole blood for COVID-19 but FDR > 0.05 for the comparator, of which 72 showed opposing effects; and three had FDR < 0.05 in the original PWAS analysis for COVID-19 but FDR > 0.05 for the comparator, all with opposing effects (Table 5; Supplementary Tables S4.3–S4.6). A cross-method concordance table for differential genes is also provided in Supplementary Table S7.2.

Effect sizes for validated differential genes were generally modest in absolute terms, consistent with the polygenic architecture of these traits. For the genes highlighted in Table 5, the absolute value of the best DDx Z-score ranged from approximately 4 to 12 across MAGMA, TWAS, and spTWAS layers, with the strongest signal at interferon-related loci (IFNAR2: best DDx Z = 12.43 in spTWAS, A2 vs. COPD; see Table 5, footnote d for interpretation of directional signs across methods). For completeness, complementary multi-trait conditional analyses using mtCOJO, which isolate COVID-19-specific signals after simultaneously conditioning on all respiratory comparators, are summarized in Table 6 and discussed in detail in Section 2.4.

PheWAS analysis demonstrated that most differential genes had been linked to measures of pulmonary function and/or immune response. Biological processes associated with differential GWASs were further explored using MAGMA gene-set analysis; we particularly focused on gene sets significant for COVID-19 only (FDR < 0.05) (Table 7A; Supplementary Table S4.7). We highlight some of the pathways below. The “DEFECTIVE_CSF2RB_CAUSES_SMDP5” pathway, identified in 11 of 24 pairwise differential analyses, highlights the distinct role of alveolar macrophages in COVID-19. The “RESPONSE_TO_TYPE_III_INTERFERON” gene set was detected in six A2-related analyses (A2 vs. asthma/COPD/pneumonia (all)/bacterial pneumonia/viral pneumonia/influenza), indicating its role in distinguishing severe COVID-19 from other respiratory conditions. The A2 vs. asthma and B2 vs. asthma comparisons identified the gene set “TYPE_I_INTERFERON_INDUCTION_AND_SIGNALING” as unique to severe and hospitalized COVID-19, whereas C2 vs. asthma revealed enrichment of “NEGATIVE_REGULATION_OF_TYPE_II_INTERFERON_PRODUCTION”.

Besides, we also performed an exploratory drug enrichment (over-representation) analysis using WebGestalt (v0.4.6) and EnrichR (https://maayanlab.cloud/Enrichr/, accessed on 1 March 2026) (Supplementary Table S4.10 and S4.11), based on the significant COVID-specific genes from MAGMA-DDx and TWAS-DDx identified in the comparisons of COVID-19 vs. flu/pneumonia (as extracted from Supplementary Tables S4.3 and S4.4). For example, some top enriched drugs included interferons, MMR vaccine, and ribavirin. These drugs were supported by several previous studies and may improve COVID-19 outcomes [34,35,36,37], although such evidence remains tentative and requires further studies.

Tissue and cell-type specificity analyses were also conducted using FUMA (Table 7B; Supplementary Tables S4.8 and S4.9). The involvement of lung and spleen tissue was demonstrated as specific to hospitalized COVID-19 (B2) when compared to (bacterial/viral) pneumonia or influenza. No cell types were identified as uniquely enriched for COVID-19.

### 2.4. Multi-Trait Differentiation at the SNP and Gene Levels

In mtCOJO analysis, the number of genome-wide significant SNPs decreased after conditioning, consistent with pleiotropy between disorders. However, 20 of the 2702 significant SNPs from the unadjusted severe COVID-19 (A2) became more significant after conditioning, forming a risk locus near *MAPT*. Similarly, five SNPs in the hospitalized COVID-19 (B2) showed increased significance after conditioning, forming a locus near *CCR3* (Table 6A). Conversely, all SNPs in the reported COVID-19 (C2) showed decreased significance, indicating broad pleiotropy.

Genes detected by at least three of the four approaches (MAGMA-mtCOJO, TWAS-mtCOJO, spTWAS-mtCOJO, and PWAS-mtCOJO) were considered prioritized/validated genes associated with the conditioned traits. The conditional analyses of A2, B2, and C2 revealed four, two, and two validated differential genes, respectively (Table 2).

Among four significant associations of severe COVID-19 detected by MAGMA-mtCOJO, *FYCO1* and *HCN3* showed greater significance in the conditional analysis compared to the unadjusted analysis (Table 6B). Further TWAS-mtCOJO analysis shows that lower genetically predicted expression of *HCN3* in lung and whole blood tissues was associated with increased risk of severe COVID-19. Specifically, this association strengthened in the conditional A2 analysis. These findings suggest that *FYCO1* and *HCN3* may be uniquely linked to severe COVID-19 rather than general respiratory diseases. Finally, MAGMA gene-set and tissue-property analyses identified two gene sets and three tissues that only reached significance after conditioning, further refining the COVID-19-specific genetic signature (Supplementary Tables S5.7 and S5.8; Table 7B). Across both shared and differential analyses, alternative splicing emerges as an important molecular mechanism linking COVID-19 to other respiratory disorders. Key splicing signals from spTWAS-cofdr (shared) and spTWAS-DDx (differential) analyses are compiled in Table 8.

A comprehensive summary of the core shared and COVID-19-specific findings across all multi-omics analyses is provided in Table 9.

**Table 6:** COVID-19-specific signals from multi-trait conditional analysis (mtCOJO). (**A**) Lead SNPs with increased significance after conditioning on respiratory traits. (**B**) Validated COVID-specific genes across omics layers.

(**A**)									
**COVID Phenotype**	**Lead SNP**	**Chr:Position**		**Nearest Gene(s)**	**β_cond**	**P_cond**	**β_unadj**	**P_unadj**	**PheWAS**
A2 (severe)	rs17652337	17:44083323		*MAPT*	−0.20	4.7 × 10^−12^	−0.12	1.2 × 10^−11^	Resp, Imm
B2 (hospitalized)	rs1491957	3:46274960		*CCR3*	0.1	3.0 × 10^−11^	0.05	3.8 × 10^−10^	Imm
(**B**)									
**COVID Phenotype**	**Gene**	**Layer**	**Tissue**	**Z_cond**	**FDR_cond**	**Z_unadj**	**FDR_unadj**	**Stronger? ^a^**	**PheWAS**
A2	*FYCO1*	MAGMA	—	—	3.6 × 10^−8^	—	3.7 × 10^−7^	**✓**	Imm
		spTWAS	Lung	4.85	1.9 × 10^−3^	7.35	5.0 × 10^−10^	—	
	*HCN3*	MAGMA	—	—	4.3 × 10^−3^	—	5.1 × 10^−3^	**✓**	Resp, Imm
		TWAS	Lung	−4.87	4.3 × 10^−3^	−4.23	1.1 × 10^−2^	**✓**	
		TWAS	Blood	−4.87	1.9 × 10^−3^	−4.23	1.0 × 10^−2^	**✓**	
	*IFNAR2*	MAGMA	—	—	1.5 × 10^−6^	—	3.7 × 10^−7^	—	—
		spTWAS	Lung	8.63	6.6 × 10^−14^	13.26	3.0 × 10^−36^	—	
	*ICAM5*	MAGMA	—	—	8.6 × 10^−4^	—	5.2 × 10^−10^	—	Resp, Imm
		PWAS	—	5.03	6.1 × 10^−4^	−5.60	2.7 × 10^−5^	**✓**	
B2	*NXPE3*	MAGMA	—	—	1.3 × 10^−3^	—	1.6 × 10^−8^	—	Imm
	*OAS1*	MAGMA	—	—	5.5 × 10^−3^	—	6.9 × 10^−10^	—	Imm
		spTWAS	Lung	4.69	3.5 × 10^−3^	7.47	3.0 × 10^−10^	—	
C2	*NXPE3*	MAGMA	—	—	8.1 × 10^−6^	—	4.5 × 10^−7^	—	Imm
	*ZBTB11*	MAGMA	—	—	1.4 × 10^−5^	—	4.5 × 10^−7^	—	Imm

^a^ **✓** indicates the gene’s association with COVID-19 became more significant after conditioning on all genetically correlated respiratory traits, suggesting COVID-19 specificity independent of shared respiratory genetics. Dashes indicate the association remained significant but did not increase. Genes validated by ≥3 of 4 omics approaches. Conditioning was performed simultaneously on all genetically correlated respiratory traits using mtCOJO (see Materials and Methods Section). TWAS and spTWAS were performed across 49 GTEx tissues; only lung and whole blood results are shown here for clarity. Complete results in Supplementary Tables S5.3–S5.6.

**Table 7:** COVID-discriminating pathways and tissue enrichments. (**A**) Gene sets enriched specifically in COVID-19 differential GWASs when compared to other respiratory infections (MAGMA gene-set analysis on DDx-derived GWASs). (**B**) Tissues enriched specifically in COVID-19 differential or conditional GWASs (MAGMA gene-property analysis).

(**A**)											
**Pathway/Gene Set**		**Source**	**N ^a^**	**COVID Pheno.**		**Best FDR_DDx (Comparison)**		**FDR_COVID ^b^**	**FDR_Resp ^b^**		**Biological Theme**
***Type I/general interferon signaling***											
Overview of IFN-mediated signaling		WP	4	A2		3.4 × 10^−5^ (A2 vs. Pneu.)		7.6 × 10^−4^	0.95		Type I IFN
Regulation of IFNA/IFNB signaling		REACTOME	5	A2, B2		2.9 × 10^−4^ (A2 vs. Pneu.)		2.7 × 10^−3^	0.88		Type I IFN regulation
Interferon alpha/beta signaling		REACTOME	4	A2		2.6 × 10^−3^ (A2 vs. Pneu.)		0.023	0.95		Type I IFN
IFNA pathway		BIOCARTA	2	A2		6.5 × 10^−3^ (A2 vs. Bact. pneu.)		0.023	0.89		Type I IFN
IFN-mediated signaling pathway		GOBP	2	A2		3.7 × 10^−2^ (A2 vs. Bact. pneu.)		0.095	0.99		IFN (general)
Interferon receptor activity		GOMF	2	A2		2.1 × 10^−2^ (A2 vs. Pneu.)		0.037	0.98		IFN receptor
***Type III interferon signaling***											
Response to type III interferon		GOBP	4 ^c^	A2		4.7 × 10^−3^ (A2 vs. Bact. pneu.)		0.014	0.95–0.97		Type III IFN
Type III interferon signaling		WP	2	A2		1.5 × 10^−2^ (A2 vs. Bact. pneu.)		0.037	0.86–0.99		Type III IFN
***GM-CSF/alveolar macrophage/surfactant***											
Defective CSF2RB causes SMDP5		REACTOME	**9** ^d^	A2, B2, C2		1.4 × 10^−5^ (B2 vs. Flu)		2.8 × 10^−5^	0.69–0.97		GM-CSF/macrophage
Diseases assoc. with surfactant metabolism		REACTOME	**12**	A2, B2, C2		1.7 × 10^−5^ (B2 vs. Flu)		2.7 × 10^−4^	0.44–0.98		Surfactant dysfunction
Surfactant homeostasis		GOBP	3	A2		1.9 × 10^−2^ (A2 vs. Viral pneu.)		0.021	0.74–1.00		Type II AEC
Alveolar lamellar body		GOCC	1	A2		3.8 × 10^−2^ (A2 vs. Viral pneu.)		0.065	1		AEC organelle
***Innate immune/antimicrobial response***											
Immune response to tuberculosis		WP	**7**	A2, B2		1.1 × 10^−3^ (A2 vs. Bact. pneu.)		7.0 × 10^−4^	0.92–0.99		Innate immunity
Eosinophils pathway		BIOCARTA	2	B2		2.4 × 10^−2^ (B2 vs. Viral pneu.)		5.4 × 10^−6^	0.88–0.97		Eosinophil biology
Measles virus infection		WP	1	B2		3.3 × 10^−2^ (B2 vs. Viral pneu.)		0.024	0.94		Antiviral response
***Other notable COVID-specific pathways***											
Multivesicular body lumen		GOCC	3	A2		3.3 × 10^−2^ (A2 vs. Flu)		0.082	0.83–0.99		Endosomal trafficking
Lung fibrosis		WP	2	A2		1.9 × 10^−2^ (A2 vs. Viral pneu.)		0.24	0.95–0.97		Fibrotic remodeling
1q21 amplicon (breast cancer)		NIKOLSKY	**12**	A2, B2, C2		1.1 × 10^−5^ (A2 vs. Flu)		6.5 × 10^−6^	0.84–0.99		Epidermal barrier ^e^
(**B**)											
**Tissue**	**Analysis**	**Comparison(s)**			**COVID Phenotype**		**Sig. in Unadj. COVID GWAS?**			**Sig. in Resp. GWAS?**	
Lung	DDx	B2 vs. Pneumonia, B2 vs. Bact. pneu., B2 vs. Influenza			B2		Yes			No	
Spleen	DDx	B2 vs. Pneumonia, B2 vs. Bact. pneu., B2 vs. Viral pneu., B2 vs. Influenza			B2		**No**			No	
Lung	mtCOJO	A2 conditioned			A2		Yes			—	
Spleen	mtCOJO	A2 conditioned			A2		**No**			—	
Thyroid	mtCOJO	A2 conditioned			A2		**No**			—	

(**A**) ^a^ Number of pairwise COVID vs. respiratory comparisons in which the gene set reached FDR_DDx < 0.05 while FDR_Resp > 0.05 (non-significant in the respiratory comparator). ^b^ FDR_COVID and FDR_Resp shown for the comparison with the best FDR_DDx. FDR_COVID reflects significance in the original COVID-19 GWAS; FDR_Resp reflects significance in the original respiratory comparator GWAS. ^c^ In the provided Supplementary Data, the manuscript text reports this pathway in 6 A2 comparisons (including A2 vs. asthma and A2 vs. COPD, which may appear in separate Supplementary Files). ^d^ Nine comparisons are presented in the provided Supplementary Data; the manuscript text reports 11 of 24 comparisons. ^e^ The 1q21 amplicon gene set contains *LCE3* cluster genes involved in epidermal differentiation and barrier function; these genes have also been associated with psoriasis and atopic dermatitis, suggesting a COVID-specific epithelial barrier disruption pathway. AEC, alveolar epithelial cell; IFN, interferon. (**B**) Bold **No** highlights tissues that emerge as significant only after differential or conditional analysis; i.e., they were masked in the original unadjusted GWAS and represent newly identified COVID-discriminating tissue signals. The spleen finding is consistent with clinical reports of splenic white pulp depletion and immune cell apoptosis in COVID-19 patients (see main text).

**Table 8:** Alternative splicing signals shared with or differentiating COVID-19 and respiratory disorders. (**A**) Shared splicing signals between COVID-19 and respiratory disorders (spTWAS-cofdr). (**B**) Differential splicing signals distinguishing COVID-19 from other respiratory disorders (spTWAS-DDx).

(**A**)							
**Gene**	**Best Comparison**	**Tissue**	**π_TT**	**Z_COVID (FDR)**	**Z_Resp (FDR)**	**Dir. ^a^**	**Biological Function**
***Transcriptional/epigenetic control***							
*MED24*	A2–Asthma	Lung	1	−4.83 (6.2 × 10^−4^)	10.61 (3.7 × 10^−23^)	↑↓	Mediator complex; RNA pol II regulation
*INTS12*	A2–Asthma	Lung	1	−4.69 (1.1 × 10^−3^)	5.76 (1.7 × 10^−6^)	↑↓	Integrator complex; snRNA processing
*NCOR1*	B2–Asthma	Lung	0.99	−4.49 (2.4 × 10^−3^)	−6.09 (2.9 × 10^−7^)	↑↑	Nuclear co-repressor; macrophage inflammation
*CCHCR1*	A2–Asthma	Blood	1	5.15 (2.1 × 10^−4^)	−6.34 (6.1 × 10^−8^)	↑↓	HLA region; psoriasis candidate gene
*TCF19*	B2–Asthma	Lung	1	−5.79 (8.3 × 10^−6^)	6.29 (9.0 × 10^−8^)	↑↓	Cell proliferation; MHC class III region
*BPTF*	A2–Asthma	Lung	0.93	−3.76 (3.0 × 10^−2^)	−3.46 (1.6 × 10^−2^)	↑↑	Chromatin remodeling (NURF complex)
***DNA repair/genomic stability***							
*ZSWIM7*	B2–Asthma	Lung	0.99	−4.53 (2.2 × 10^−3^)	−6.08 (2.9 × 10^−7^)	↑↑	Homologous recombination; DNA repair
***Innate immunity/fibrosis***							
*ATP11A*	B2–IPF	Lung	1	−6.79 (3.1 × 10^−8^)	6.26 (7.2 × 10^−7^)	↑↓	Phospholipid flippase; pulmonary fibrosis
*DPP9*	A2–IPF	Lung	0.99	−6.94 (8.2 × 10^−9^)	−4.09 (1.8 × 10^−2^)	↑↑	Dipeptidyl peptidase; COVID–IPF locus
*TRIM4*	A2–IPF	Lung	0.99	−3.96 (1.6 × 10^−2^)	−4.69 (2.0 × 10^−3^)	↑↑	E3 ubiquitin ligase; IFN-β induction
*BAG6*	A2–IPF	Lung	0.98	3.72 (3.4 × 10^−2^)	4.46 (4.7 × 10^−3^)	↑↑	Co-chaperone; NK cell cytotoxicity
*NPNT*	A2–Asthma	Lung	1	−6.20 (5.2 × 10^−7^)	8.70 (2.2 × 10^−15^)	↑↓	Nephronectin; integrin–ECM signaling
***Airway inflammation***							
*GSDMB*	B2–COPD	Lung	1	−4.75 (1.0 × 10^−3^)	6.13 (3.9 × 10^−6^)	↑↓	Gasdermin B; pyroptosis; inflammation
*MUC1*	C2–Asthma	Lung	1	6.55 (1.8 × 10^−7^)	−4.72 (2.2 × 10^−4^)	↑↓	Mucin 1; epithelial barrier defense
***Metabolic/mitochondrial***							
*SURF1*	C2–COPD	Blood	0.99	6.98 (1.5 × 10^−8^)	3.99 (1.4 × 10^−2^)	↑↑	Cytochrome c oxidase assembly
(**B**)							
**Gene**	**Best Comparison**	**Tissue**	**Z_DDx**	**FDR_DDx**	**Z_COVID (FDR)**	**Z_Resp (FDR)**	**Biological Function**
***Interferon receptor regulation***							
*IFNAR2*	A2 vs. COPD	Lung	12.43	1.8 × 10^−31^	13.26 (3.0 × 10^−36^)	−0.75 (0.86)	Type I IFN receptor subunit
*OAS1*	A2 vs. Bact. pneu.	Lung	6.51	1.0 × 10^−7^	6.57 (6.8 × 10^−8^)	0.12 (1.00)	2′–5′ Oligoadenylate synthetase
*TYK2*	A2 vs. IPF	Lung	−6.49	2.1 × 10^−7^	−6.77 (2.5 × 10^−8^)	2.51 (0.44)	JAK–STAT signaling kinase
***Vascular/endothelial biology***							
*ARHGAP27*	A2 vs. Pneumonia	Lung	−6.67	4.0 × 10^−8^	−6.53 (8.8 × 10^−8^)	1.46 (0.90)	Rho GTPase; endothelial barrier
*RASIP1*	A2 vs. Pneumonia	Lung	−6.87	1.1 × 10^−8^	−6.67 (4.3 × 10^−8^)	1.97 (0.81)	Endothelial junction assembly
***Extracellular matrix/tissue remodeling***							
*THBS3*	A2 vs. Pneumonia	Lung	−9.25	6.1 × 10^−17^	−9.28 (4.8 × 10^−17^)	0.31 (0.99)	Thrombospondin 3; ECM
*MTX1*	A2 vs. COPD	Lung	−7.59	7.7 × 10^−11^	−7.28 (7.9 × 10^−10^)	2.42 (0.30)	Mitochondrial protein import
***Autophagy/intracellular trafficking***							
*FYCO1*	A2 vs. IPF	Lung	5.25	1.8 × 10^−4^	7.35 (5.0 × 10^−10^)	−0.52 (0.95)	Autophagosome transport
*PLEKHM1*	A2 vs. Pneumonia	Lung	−6.79	1.8 × 10^−8^	−6.65 (4.7 × 10^−8^)	1.44 (0.90)	Autophagy–lysosomal trafficking
***Inflammatory cell death***							
*GSDMB*	B2 vs. Pneumonia	Lung	5.09	2.7 × 10^−4^	4.75 (1.0 × 10^−3^)	−2.18 (0.73)	Gasdermin B; pyroptosis
***Other notable signals***							
*FBRSL1*	B2 vs. Asthma	Lung	−5.33	3.6 × 10^−5^	−4.55 (2.2 × 10^−3^)	2.72 (0.09)	Fibrosin-like 1; chromatin regulation
*SACM1L*	A2 vs. COPD	Lung	6.13	1.0 × 10^−6^	5.42 (3.9 × 10^−5^)	−2.84 (0.17)	Phosphoinositide phosphatase
*SCAMP3*	A2 vs. Influenza	Blood	−4.42	5.5 × 10^−3^	−4.16 (1.2 × 10^−2^)	2.13 (0.94)	Secretory carrier membrane protein
*TMEM116*	A2 vs. COPD	Lung	−4.65	1.1 × 10^−3^	−3.60 (4.6 × 10^−2^)	3.31 (0.08)	Transmembrane protein
*DPP9*	A2 vs. COPD	Lung	−6.81	2.1 × 10^−8^	−6.94 (8.2 × 10^−9^)	1.23 (0.72)	Dipeptidyl peptidase

^a^ Panel (**A**) ↑↑ = concordant direction; ↑↓ = discordant direction. In Panel (**B**), all listed genes show significant splicing association with COVID-19 (FDR < 0.01) but not with the respiratory comparator (FDR > 0.05), confirming COVID-discriminating splicing regulation. The strongest result per gene is shown; many genes appear across multiple comparisons (see Supplementary Tables S2.5 and S4.5 for complete results). For example, *IFNAR2* is differential in A2 vs. asthma/ILD/IPF/COPD/pneumonia; *THBS3* in A2 vs. IPF/COPD/pneumonia/influenza; *MED24* is shared across all three COVID phenotypes with asthma and COPD. Notably, some genes appear in both panels across different trait comparisons (e.g., *GSDMB* is shared between B2 and COPD but differential between B2 and pneumonia; *DPP9* is shared between A2 and IPF but differential between A2 and COPD/pneumonia), illustrating that the same gene’s splicing regulation may contribute to both shared and distinct pathobiology depending on the respiratory condition being compared.

**Table 9:** Summary of core shared pan-respiratory and COVID-19-specific findings across multi-omics layers.

Functional Theme	Representative Genes	Supporting Analytical Methods	Brief Biological Role
***Shared Pan-Respiratory Axis***			
Pyroptosis and inflammatory cell death	*GSDMA*, *GSDMB*	MAGMA-cofdr, spTWAS-cofdr, gwas-pw	Gasdermin-mediated pore formation shared across COVID-19, asthma, and COPD
Innate immunity and fibrosis	*DPP9*, *ATP11A*	MAGMA-cofdr, TWAS-cofdr, spTWAS-cofdr, hyprcoloc	Dipeptidyl peptidase and lipid flippase with shared COVID-19/IPF signals; ATP11A shows opposing effects between COVID-19 and IPF
MHC and antigen presentation	*TCF19*, *MICB*, *CCHCR1*	MAGMA-cofdr, TWAS-cofdr, spTWAS-cofdr	Immune regulation within the chromosome 6p21 MHC region ^a^
Leukocyte adhesion and migration	*ICAM1*, *ICAM5*	MAGMA-cofdr, PWAS-cofdr	Cell adhesion molecules mediating leukocyte transendothelial migration
Transcriptional co-regulation	*MED24*, *NCOR1*	MAGMA-cofdr, TWAS-cofdr, spTWAS-cofdr	Mediator complex subunit and nuclear co-repressor regulating macrophage inflammatory gene expression
***COVID-19-Specific Axis***			
Type I and III interferon signaling	*IFNAR2*, *OAS1*, *TYK2*	MAGMA-DDx, spTWAS-DDx, CC-GWAS	Interferon receptor subunits and JAK-STAT kinase; genetically elevated in severe COVID-19 but not in other respiratory infections
GM-CSF and alveolar macrophage function	*CSF2RB pathway* ^b^	MAGMA-DDx pathway enrichment	GM-CSF receptor signaling driving alveolar macrophage maturation and surfactant clearance
Surfactant metabolism	*SFTPB*, *SFTPC pathway* ^b^	MAGMA-DDx pathway enrichment	Pulmonary surfactant proteins required for alveolar surface tension regulation
Autophagy and intracellular trafficking	*FYCO1*	MAGMA-DDx, spTWAS-DDx, mtCOJO	Autophagosome transport factor; association with severe COVID-19 strengthened after conditioning on shared respiratory genetics
Ion channel regulation	*HCN3*	MAGMA-DDx, TWAS-mtCOJO	Hyperpolarization-activated cyclic nucleotide-gated channel; association strengthened after conditioning

^a^ Genes in the HLA/MHC region require caution in interpretation due to complex LD and high gene density (see Section 4.3.1). ^b^ These rows reflect pathway-level enrichment findings. The specific genes listed are representative members of the enriched pathway rather than individually validated genes from gene-level analyses. Genes listed are representative examples; complete results are in Supplementary Tables S2.3–S2.6 and S4.3–S4.6.

## 3. Discussion

### 3.1. Overview

Here we disentangled shared and disease-discriminating (differential) genetic contributions between COVID-19 outcomes (A2/B2/C2) and eight respiratory risk factors/disorders using a unified summary-statistics framework across 24 pairwise and three multi-trait comparisons, at a multi-omics level (Figure 1). Rather than evaluating “genetic overlap” per se, we explicitly decomposed the genetic architecture into: (i) shared association signals (variants/genes affecting multiple traits) and (ii) differential signals (variants/genes showing heterogeneous effects between COVID-19 and related respiratory phenotypes). By integrating SNP-level association patterns with multi-omics gene-level evidence (MAGMA, TWAS, spTWAS, and PWAS), we moved from the SNP level to mechanistically interpretable hypotheses about various molecular layers, including expression, splicing, and protein abundance, through which genetic risk may act.

This work complements but is distinctly different from other studies focused on revealing genetic factors for individual respiratory infections or disorders. The current study, for example, is fundamentally different from our recent GWAS on hospitalized influenza [38], which focused on discovering specific influenza risk loci (e.g., *ST6GAL1*) and genetic factors underlying time to hospitalization. The present study treats influenza GWAS statistics as just one component of a broader analysis, where COVID-19 serves as the index trait. In addition, whereas the influenza study only included a single pairwise comparison, the current work encompasses 24 pairwise and 3 multi-trait comparisons involving three COVID-19 phenotypes (A2/B2/C2; only B2 was covered in Ref. [38]) and a wide spectrum of eight respiratory disorders. We employed multiple complementary methods for both shared (cofdr, gwas-pw, and hyprcoloc) and differential (DDx, CC-GWAS, mtCOJO) signal detection and systematically integrated multi-omics layers. The multi-trait conditional analysis (mtCOJO), which isolates COVID-specific signals, is also unique to this study. Here, our objective is not to characterize individual respiratory infections as in Ref. [38] or other GWASs, but rather to apply a unified framework to systematically disentangle the shared “pan-respiratory” genetic backbone from the specific molecular mechanisms (e.g., splicing defects, interferon dysregulation) that distinguish COVID-19 from the wider spectrum of respiratory diseases.

### 3.2. Novelty of the Proposed Analytic Framework

Our study applies a framework that moves beyond standard genetic overlap approaches in three ways (Figure 1). First, prior methods such as global genetic correlation and overlap of genome-wide significant loci do not provide locus-level posterior probabilities, cannot distinguish between statistical sharing and shared causal mechanisms, and do not quantify trait-discriminating effects. cofdr addresses the first limitation by scanning genome-wide, while coloc-type algorithms (gwas-pw and hyprcoloc) provide complementary locus-level colocalization support. DDx and CC-GWAS then identify variants with divergent effects between traits, a question that shared-signal methods cannot answer.

Second, by extending the analysis to TWAS, spTWAS, and PWAS, the framework allows the data to indicate at which molecular layer (expression, splicing, or protein abundance) a shared or differential signal operates. To our knowledge, this is the first study to examine both shared and distinct genetic architecture of respiratory diseases across all three layers simultaneously.

Third, multi-trait conditioning via mtCOJO isolates signals that remain associated with COVID-19 after accounting for all tested respiratory traits simultaneously, a step that pairwise approaches cannot achieve.

Throughout this manuscript, we adhere to the terminology formally defined in Section 4.3.1 to distinguish among shared statistical signals (cofdr), colocalized signals (gwas-pw, hyprcoloc), pleiotropic effects, and candidate causal gene nominations. Furthermore, as detailed in Section 4.5.3, we apply the term “convergently supported” specifically to describe genes identified by three or more omics approaches.

### 3.3. Revealing “Differential” Signals That Distinguish COVID-19 from Other Respiratory Diseases

Our systematic genome-wide scan identified a refined set of biologically coherent differential signals that distinguish COVID-19 from other respiratory diseases, beyond what can be inferred from global genetic correlation or overlap of genome-wide significant loci. Across pairwise comparisons, we detected 214 differential loci, including 48 loci with opposite allelic directions between COVID-19 and a comparator trait. Our findings highlighted not only shared susceptibility but also genetic trade-offs (protective for one disorder but deleterious for another) across COVID-19 and other respiratory conditions. Below we highlight some of our important findings. We note that individual differential signals, while statistically robust, typically reflect modest effect sizes consistent with the polygenic nature of complex traits. Their biological significance derives primarily from convergence across multiple independent comparisons and analytical layers rather than from any single large-effect variant. Experimental validation remains necessary to confirm the functional relevance of specific candidates.

### 3.4. Pathways Distinguishing COVID-19 from Other Respiratory Infections

A key question regarding COVID-19 pathology is as follows: what are the unique mechanisms that drive COVID-19 pathophysiology when compared to similar acute respiratory infections such as pneumonia and influenza? Using a case–case GWAS approach, our differential loci (DDx) and corresponding gene-set analysis (MAGMA-DDx) isolated genomic signals that are more strongly associated with SARS-CoV-2 than other respiratory infections (Table 7A; Supplementary Table S4.7).

#### 3.4.1. Dysregulated Type I and Type III Interferon Responses Underlying Severe COVID-19

Across comparisons of COVID vs. pneumonia and COVID vs. influenza, the most recurrent enrichments converge on interferon biology, including BIOCARTA_IFNA_PATHWAY, REACTOME_INTERFERON_ALPHA_BETA_SIGNALING, REACTOME_REGULATION_OF_IFNA_IFNB_SIGNALING, GOBP_INTERFERON_MEDIATED_SIGNALING_PATHWAY, and GOBP/WP_TYPE_III_INTERFERON_SIGNALING.

These findings suggest the genetic architecture of severe COVID-19 is defined by a specific dysregulation of Type I and Type III interferon responses, concordant with previous reports which highlighted the importance of these pathways [39,40,41,42]. Importantly, interferons have also been investigated in clinical trials. Type III interferons, specifically peginterferon lambda-1a, showed promising results in a recent randomized controlled trial [36]. In predominantly vaccinated outpatients, a single subcutaneous dose within 7 days of symptom onset reduced the risk of hospitalization or emergency room visits by ~51%. Our results align with prior evidence pointing to interferon dysregulation in severe COVID-19. They show how genetic data can help prioritize targets for drug discovery or repurposing, though these findings do not establish therapeutic efficacy.

In summary, our DDx analysis indicates that a dysfunctional interferon-mediated (Type I/III) antiviral response is a potential defining genetic mechanism underlying severe COVID-19 compared to other respiratory infections. These pathway enrichments also provide functional context for the strong splicing-based differential signals at interferon-related genes (e.g., *IFNAR2* in spTWAS-DDx).

#### 3.4.2. Alveolar Epithelial, Macrophage, and Surfactant Biology in COVID-19

A second converging theme in the COVID-versus-pneumonia/flu differential pathway results is alveolar epithelial and surfactant biology. Related pathways included, for example, GOBP_SURFACTANT_HOMEOSTASIS, GOCC_ALVEOLAR_LAMELLAR_BODY, and REACTOME_DISEASES_ASSOCIATED_WITH_SURFACTANT_METABOLISM. These enrichments highlight the role of Type II alveolar epithelial cell function and the surfactant system in COVID-19, consistent with the clinical and pathological findings of diffuse alveolar injury in severe COVID-19 [43,44].

Additionally, we identified “Defective CSF2RB causes Pulmonary Surfactant Metabolism Dysfunction (SMDP5)” as a pathway showing enrichment in the differential analysis, which suggests failure of surfactant homeostasis and alveolar macrophage dysfunction as a COVID-specific mechanism. *CSF2RB* is the receptor for GM-CSF, a cytokine critical for the maturation of alveolar macrophages and their ability to clear surfactants from the lungs [45]. Our findings suggest that genetic predisposition to severe COVID-19 is partially due to a distinct failure in the GM-CSF/alveolar macrophage axis, leading to the accumulation of cellular debris and surfactant dysregulation, also typical of acute respiratory distress syndrome (ARDS).

Notably, GM-CSF-based treatments (administration or inhibition) have been tested in clinical trials for respiratory infections including COVID-19. Some trials showed protective effects for COVID-19 [46,47], though the overall results were mixed [48]. Our study is consistent with the notion that GM-CSF signaling may represent a COVID-19-specific therapeutic target relative to other respiratory infections, though confirmation of clinical benefit requires further randomized clinical trial evidence.

### 3.5. Differential Tissue Involvement in COVID-19 Versus Other Respiratory Infections

Our tissue enrichment analysis of differential GWASs revealed that genes specifically associated with hospitalized COVID-19 (B2), but not with pneumonia or influenza, were enriched for expression in the lung and spleen (Table 7B). While lung involvement is expected, the spleen-specific enrichment is notable. Studies have shown that splenic white pulp, responsible for immune cell production, is significantly reduced in COVID-19 patients [49]. Another study has also revealed a high rate of apoptosis of immune cells in the spleen in COVID-19 [50]. Our work provides genetic support for clinical observations of splenic pathology in COVID-19 patients, suggesting such pathology may be more specific to COVID-19 compared to other respiratory infections. This tissue-specific signature may reflect the ability of SARS-CoV-2 to cause systemic immune dysregulation beyond the respiratory tract.

### 3.6. FYCO1 and HCN3 as Potential COVID-19-Specific Risk Genes

Our multi-trait conditional analysis (mtCOJO) further identified genetic variants that influence COVID-19 risk independently of shared respiratory disease biology. Our analysis revealed several genes, including *FYCO1* and *HCN3*, as tentative COVID-19-specific genes whose association with critical COVID-19 (A2) became more significant after conditioning on other respiratory disorders (Table 6B).

*FYCO1* is a protein essential for autophagosome transport along microtubules. Although the gene is located within a known COVID-19 risk locus (3p21.31), our conditional analysis reveals that this gene might be specific to COVID-19 pathology. This suggests that autophagy pathways may play a key role in mediating COVID-19 risks.

*HCN3* (hyperpolarization-activated cyclic nucleotide-gated channel 3) is the only gene that remains significant in TWAS after conditioning, with a stronger effect size after adjustment. This gene encodes a voltage-gated ion channel, largely studied in cardiac and neuronal tissues. Interestingly, HCN channels have recently been implicated in the neuronal regulation of smooth muscle tone of the airway [51].

### 3.7. Alternative Splicing: An Understudied Mechanism Underlying Shared and Distinct Genetic Architecture

Alternative splicing has been proposed as an important mechanism underlying human diseases, but it was relatively understudied in respiratory disorders, including COVID-19. An exception was a recent work by Nakanishi et al. [30], who utilized Mendelian randomization to establish the causal role of splicing in genes such as *NPNT*, *ATP11A*, *OAS1*, and *DPP9* in severe COVID-19. As a secondary analysis, they also searched for the effects of the COVID-associated splicing QTLs on other diseases using the Open Targets Genetics database. However, the scope and objectives of our study differ significantly from Nakanishi et al. Here, we conducted a systematic genome-wide scan of shared and distinct genetic architecture of COVID-19 with a wide array of respiratory disorders, using a multi-omics framework including the study of alternative splicing.

#### 3.7.1. Shared Alternative Splicing Signals

Notably, our genome-wide splicing TWAS shared-signal scan (spTWAS-cofdr) identifies additional splicing-associated genes not previously reported (Table 8A). Among the shared-signal results, the most consistent COVID-asthma signals are not limited to classical antiviral loci but include a cluster of genes with strong shared splicing association in lung and/or whole blood, such as *MED24*, *INTS12*, *TCF19*, *NCOR1*, *ZSWIM7*, *BPTF*, and *CCHCR1*, implicating the role of transcriptional control, epigenetic remodeling, and genomic stability in the shared genetic architecture of COVID-19 and asthma. Specifically, *MED24* and *INTS12* serve as bridges between signaling and RNA production [52,53]; *NCOR1* restricts macrophage-mediated inflammation [54]; and *ZSWIM7* regulates homologous recombination and DNA repair [55].

Our shared splicing signals for COVID-IPF extend beyond the well-studied ATP11A/DPP9 axis by highlighting additional candidates such as *TRIM4* and *BAG6*. *TRIM4* has been reported to regulate virus-induced interferon pathways [56,57], while *BAG6* may play a role in immune regulation by regulating NK cell activities [58]. These signals suggest that the COVID-IPF relationship may reflect shared or antagonistic regulation of innate immune responses and antigen presentation, potentially associated with hyperinflammatory responses in both diseases.

Shared splicing analysis also revealed signals connecting COVID-19 outcomes to obstructive airway disease. For example, *GSDMB* splicing was highlighted in COVID hospitalization (B2)-COPD comparisons. Notably, *GSDMB* was reported to mediate inflammatory responses in the airway following viral infection [59].

#### 3.7.2. COVID-Discriminating Splicing Signals—Interferon Receptor Regulation, Vascular Biology, and Intracellular Trafficking

Our differential spTWAS (spTWAS-DDx) results identify genes whose genetically regulated splicing signals separate COVID-19 from other respiratory disorders (Table 8B). The most prominent example is *IFNAR2*, which shows differential splicing signals across multiple comparisons (e.g., A2 vs. asthma/ILD/IPF/COPD and A2 vs. pneumonia-meta). These findings provide evidence that interferon receptor-related regulatory differences, including via alternative splicing, may be a discriminator between severe COVID-19 and other respiratory conditions.

In COVID vs. pneumonia comparisons, differential splicing involved genes such as *ARHGAP27*, *RASIP1* and *PLEKHM1*. These genes are connected to vascular/endothelial barrier function and autophagy-lysosomal biology [60,61]. Additional differential candidates such as *MTX1* and *THBS3* (seen across multiple comparisons) further suggest roles for mitochondrial/organellar stress responses and extracellular matrix/repair programs in distinguishing COVID-19 from other respiratory conditions.

It is worth noting that the alternative splicing signals identified in our study reflect germline genetic regulation of splicing via cis-sQTLs, and do not capture the additional layer of splicing regulation mediated by epigenetic mechanisms. DNA methylation and histone modifications are well-established regulators of co-transcriptional splicing [62,63], and SARS-CoV-2 infection may induce epigenetic changes that shift isoform balance, including potentially at IFNAR2 and other interferon pathway genes, through mechanisms independent of inherited genetic variation. Characterizing such infection-induced changes requires cell-type-specific epigenomic profiling and represents an important direction for future research.

### 3.8. Shared Genetic Signals Across COVID-19, Asthma, and COPD

While previous studies have focused on the genetic overlap between fibrotic lung disease and COVID-19, our systematic pairwise and multi-trait colocalization spanning eight respiratory conditions revealed a broader “pan-respiratory” genetic backbone underlying airway diseases. In particular, we identified a distinct set of genes that demonstrate pleiotropy across COVID-19, asthma, and COPD (Table 4; Supplementary Tables S2.3–2.5). These genes involve several different biological pathways, such as pyroptosis and inflammatory cell death (e.g., *GSDMA*, *GSDMB*) [64], antigen presentation and MHC signaling (*TCF19*, *MICB*) [65], leukocyte migration and cell adhesion (*ICAM1*, *ICAM5*) [66], and protein degradation (*PSMD3*) [67]. Our findings provide important insight into possible shared pathogenesis between COVID-19 and other chronic airway diseases.

Due to space constraints, this discussion highlights only a subset of the shared and differential splicing signals (as alternative splicing is relatively understudied), alongside selected genes/pathways with high scientific or clinical relevance. However, our study encompasses a comprehensive, genome-wide multi-omics analysis (MAGMA, TWAS, spTWAS, and PWAS) across more than 20 trait comparisons (with a total of >100 sets of different analyses). The complete results are detailed in the Supplementary Tables, which we believe will serve as a valuable resource for the community to further explore the unique and shared genetic architectures, molecular mechanisms, and potential therapeutic targets underpinning COVID-19 and related respiratory phenotypes.

### 3.9. Clinical Implications and Translational Potential

Our differential analysis highlights specific genes, pathways, and pathological mechanisms underlying COVID-19 as compared to other respiratory infections, offering directions for precision medicine. First, the isolation of COVID-specific pathways, including the GM-CSF/surfactant dysfunction axis and Type III interferon signaling, points toward therapeutic hypotheses beyond general anti-inflammatory approaches. Notably, drugs targeting these mechanisms, such as GM-CSF modulators and peginterferon lambda, have already entered randomized controlled trials. While our genetic findings do not directly establish clinical efficacy, they are highly consistent with the biological rationale of these trials and may help prioritize future therapeutic targets, reinforcing the translational potential of our data for drug discovery and repurposing. Further investigation into other COVID-specific signals identified in this study is warranted.

Second, the opposing effects observed in our splicing and other omics analyses may be relevant to the side effects of medications. For example, a drug that improves COVID-19 may theoretically lead to adverse effects on other respiratory conditions if the drug acts on genes with opposing effects. An example is ATP11A in COVID-19 versus IPF, which demonstrates opposite effects in our spTWAS. Nevertheless, these hypotheses will require further testing and experimental validation.

Third, identifying shared genetic risk factors provides a foundation for the development of biomarkers for long-term sequelae. Individuals harboring risk alleles at these shared loci or showing associated changes in gene expression or protein levels may be at heightened risk for both acute COVID-19 severity and pulmonary sequelae. As such, our findings also advocate for the development of integrative polygenic risk scores (PRSs) that simultaneously account for COVID-19 severity and susceptibility to respiratory comorbidities. Such scores could facilitate personalized risk stratification, forecasting not only the severity of the acute infection but also the long-term probability of post-COVID respiratory sequelae.

We emphasize, however, that the clinical implications discussed here are hypothesis-generating. Definitive assessment of causal mechanisms and therapeutic efficacy requires rigorous experimental validation and randomized clinical trial evidence.

### 3.10. Limitations and Future Directions

Several limitations are worth noting. First, findings in this study should primarily be interpreted as hypothesis-generating, rather than confirmatory. The analyses prioritize associated loci, genes, and mechanistic hypotheses, but do not establish causal genes, underlying biological mechanisms, or treatment efficacy. Confirmation of causal relationships requires functional and experimental validation, while evidence of therapeutic benefit requires randomized clinical trials.

Second, the COVID-19 HGI Release 7 summary statistics were derived from a meta-analysis of cohorts recruited across multiple countries, time periods, and pandemic waves. Each of the three COVID-19 phenotypes (A2, B2, C2) represents a composite outcome defined across contributing studies that may differ in ascertainment criteria, hospitalization thresholds, and testing access. The genetic associations identified may also be influenced by the circulating SARS-CoV-2 variant at the time of infection, vaccination status, and regional differences in pandemic intensity. These sources of heterogeneity are partially mitigated by the large sample sizes and meta-analytic design of the HGI, but cannot be fully eliminated. Future analyses stratified by vaccination status, circulating viral variant, ascertainment period, and other major clinical factors may be necessary.

Third, this study is primarily restricted to European-ancestry populations. Genetic architecture, linkage disequilibrium patterns, allele frequencies, and effect sizes of risk variants may differ substantially across ancestries, limiting the generalizability of our findings. The PWAS prediction models used here were derived from individuals of European ancestry and may not generalize to other populations. Replication in multi-ancestry cohorts is an important priority for future work.

Fourth, several respiratory traits have relatively moderate case numbers, which may reduce statistical power for both shared and differential analyses.

Fifth, the TWAS and spTWAS prediction models are derived from GTEx v8 (49 tissues, N = 838 individuals). Relevant cell types for COVID-19 and respiratory diseases, such as alveolar epithelial cells and alveolar macrophages, are not available as distinct cell-type-specific prediction models, potentially limiting the tissue-specificity of inference. In addition, prediction model accuracy (R^2^) varies across genes and tissues, and TWAS/spTWAS/PWAS associations can be confounded by nearby causal variants affecting other genes in the same LD block. These methods identify associated genes rather than causal mechanisms.

Finally, while our genome-wide multi-omics framework provides a systematic characterization of the heritable genetic architecture underlying COVID-19 and related respiratory disorders, we acknowledge that genetic predisposition represents only one dimension of a complex and multifactorial disease process. The clinical heterogeneity of COVID-19 is also shaped by epigenetic modifications, including SARS-CoV-2-induced changes in DNA methylation, histone modifications, and non-coding RNA-mediated regulation [68], as well as by microbiome composition, environmental exposures, and prior immune history. Microbiome–virus interactions and infection-induced epigenetic reprogramming, as highlighted for example by Brogna et al. [69], may further modulate host immune responses and gene expression in ways that complement and interact with heritable genetic predisposition. Future integrative studies combining genetic, epigenomic, and microbiome data will be important for a more complete understanding of COVID-19 pathogenesis.

## 4. Materials and Methods

### 4.1. GWAS Summary Statistics

#### 4.1.1. GWAS Data Harmonization

We leveraged data from the COVID-19 Host Genetics Initiative (COVID-19 HGI Release7) [15] comparing genetic structures between COVID-19 and other related respiratory conditions. GWAS summary statistics were collected for 8 respiratory disorders related to COVID-19 [70]. GWAS datasets were formatted and harmonized using MungeSumstats (v1.6.0) [71], with default parameters. Note that MungeSumstats ensures consistency of allele assignment and direction of effects across studies. Summary statistics were aligned to GRCh37, and only bi-allelic SNPs with MAF ≥ 0.01 present in the reference file (dbSNP144) were preserved.

#### 4.1.2. GWAS Meta-Analysis

A meta-analysis of pneumonia summary statistics was performed using METAL (version released on 2011-03-25) [72] between PanUKBB (phecode 480) and Finngen (J10 pneumonia) samples. To impute unmeasured SNPs, meta-analyzed variants present in both cohorts were utilized as input for DIST (v1.0.0) [73] using the 1000 Genome EUR reference panel. Follow-up studies were conducted on the imputed common variants (INFO > 0.3). Using the above workflow, we also generated meta-analyzed GWASs for bacterial pneumonia (PanUKBB phecode 480.1; Finngen J10 bacterial pneumonia), viral pneumonia (PanUKBB phecode 480.2; Finngen J10 viral pneumonia), and influenza (PanUKBB phecode 481; Finngen influenza). The effective sample sizes differ substantially across the meta-analyzed pneumonia subtypes (Table 1). The all-pneumonia (N_cases = 58,600) and bacterial pneumonia (N_cases = 22,399) meta-analyses have substantially larger case counts than the viral pneumonia (N_cases = 2373) and influenza (N_cases = 4405) meta-analyses, resulting in markedly different statistical power across these comparators. Where viral pneumonia is used as the comparator in pairwise analyses, this is noted explicitly in the relevant tables. The all-pneumonia meta-analysis was used as the primary pneumonia comparator in pathway-level and tissue-enrichment analyses given its greater statistical power.

### 4.2. SNP-Based Decorrelation

A decorrelated Z-score matrix was generated to minimize the effect of sample overlap, based on the method described in Ref. [74]:

(1) $$Z_{decor}=C^{-1/2}Z$$

where C is a matrix with ones at the diagonal and the LDSC intercept [75] as off-diagonal elements representing sample overlap, and the matrix Z contains the original Z-scores. For GWASs with unreliable LDSC estimates due to small sample sizes, sample overlap was corrected using the correlation between Z-scores of putatively null SNPs, following the approach used by the software METAL for overlapping samples [72,76]. First, strongly associated loci were removed by applying a cutoff value for absolute Z-scores (|Z| < 2). Second, the covariance matrix was derived by fitting “mle.tmvnorm” [77] with truncated Z-scores, and the corresponding correlation between the two traits was calculated. As shown in Supplementary Table S1, most of the traits studied showed only minimal or small sample overlap.

### 4.3. Shared Genetic Architecture at the SNP Level

#### 4.3.1. Pairwise Shared-Variant Analysis Using cofdr

To identify genetic variants that jointly contribute to the risk of COVID-19 and other related traits, the “cofdr” algorithm (proposed in Ref. [78], custom implementation available at https://github.com/xxxue96/ddx_cofdr_workflow, accessed on 1 March 2026) was applied with a decorrelated Z-score matrix as input. Briefly, a four-group mixture model was constructed to determine whether a genetic variant is associated with trait 1, trait 2, both traits, or neither trait 1 nor trait 2.

$$f\left(z_{A},z_{B}\right)=p_{00}f_{00}\left(z_{A},z_{B}\right)+p_{10}f_{10}\left(z_{A},z_{B}\right)+p_{01}f_{01}\left(z_{A},z_{B}\right)+p_{11}f_{11}\left(z_{A},z_{B}\right)$$

The likelihood function was maximized with the EM algorithm to estimate the posterior probability that an SNP is shared by two traits (defined as posterior probability ≥0.9 in this study), associated with trait 1 (or trait 2) only, and associated with neither trait 1 nor trait 2. These estimates can be considered as a two-dimensional extension of the standard local false discovery rate [79]. Here we allow for a highly flexible distribution of the non-null effects, based on a continuous mixture model. The distribution of z under the alternative is assumed to follow

$$f_{1}\left(z\right)=\int N(z|\mu +\theta ,\sigma^{2})g(\theta )d\theta ,$$

where g(θ) is a flexible distribution for the non-null effects. We employed the “prfdr” algorithm proposed by Scott et al. [80] to estimate the alternative density. The bivariate density is estimated by the product of the marginal densities (after the decorrelation step). The method can be readily extended to more than two traits; for example, an 8-component mixture model can be constructed for three traits. For details, please refer to Ref. [78] and the Supplementary Information therein.

cofdr also conducted a likelihood ratio test for the existence of pleiotropy at a genome-wide level, testing the null hypothesis.

**H0.**  *π11 = π1⋅ × π⋅1 (i.e., independence of association status across traits), where π11 denotes the proportion of SNPs associated with both traits, and π1⋅ and π⋅1 denote the marginal proportions associated with each trait, respectively.*

While cofdr is designed to identify shared or pleiotropic genetic signals, methods such as gwas-pw, coloc, and hyprcoloc represent alternative approaches that detect shared causal variants based on linkage disequilibrium (LD) structure. We employ a hybrid strategy here, as these two classes of methods are complementary. cofdr is highly scalable to millions of SNPs and multiple traits, enabling efficient genome-wide scanning for shared variants. In contrast, coloc-type algorithms typically focus on individual loci of interest.

Notably, cofdr does not require an LD reference panel, which reduces issues arising from ancestry mismatch between the reference panel and the study population. It also provides a likelihood ratio test for genetic overlap at a genome-wide level and requires only *p*-values (or z-scores), the most commonly available summary statistics. Consequently, cofdr can handle gene-level statistics (such as those from MAGMA, TWAS, SpTWAS, or PWAS) as input and estimate the posterior probability of shared gene-level signals. As coloc-type algorithms are primarily designed for SNP-level data, there is currently a lack of studies investigating shared signals at the gene level across multiple molecular layers. On the other hand, coloc-type algorithms are specifically suited to determine whether two traits share the same causal variant at a specific locus while explicitly accounting for LD information and therefore serve a complementary objective.

It is important to distinguish between several related but conceptually distinct terms used throughout this study. A shared statistical signal, as identified by cofdr, indicates that a genomic region or variant shows association with both traits simultaneously. This pattern does not imply a single shared causal variant or establish an underlying biological mechanism. Colocalization, assessed by gwas-pw and hyprcoloc, provides evidence that the same causal variant underlies the associations in both traits within a given linkage disequilibrium-independent region. Pleiotropy refers to a variant or gene influencing multiple phenotypes, which may occur through the same or biologically distinct pathways. Causal gene identification is not achievable through any of the summary-statistics-based methods employed here and requires experimental validation. These interpretive distinctions apply across all analytical layers.

Several caveats are worth mentioning. We note that within the HLA/MHC region (chromosome 6p21), extensive linkage disequilibrium, high gene density, and overlapping signals make causal inference especially challenging. Therefore, gene-level annotations in this locus must be interpreted with caution and treated strictly as candidates requiring further fine-mapping and experimental follow-up. Similarly, for TWAS and spTWAS, the gene mapped to a significant eQTL or sQTL signal may not be the effector transcript, as a neighboring gene in linkage disequilibrium may drive the true causal signal. For MAGMA, physical proximity of a SNP to a gene boundary does not automatically establish functional relevance.

#### 4.3.2. Prioritization of Shared Genetic Variants Using gwas-pw and hyprcoloc

Shared SNPs identified from cofdr were further prioritized with gwas-pw (v0.21) [81] and hyprcoloc (v1.0) [82]. gwas-pw computes the posterior probability of association (PPA) for pairwise comparisons in which LD-independent genomic regions [83] are implicated in only one trait (models 1 and 2), both traits (model 3), or both traits with different variations (model 4). Correlations estimated by “mle.tmvnorm” or LDSC intercepts were used as correction factors in overlapping cohorts. A putative causal variant can then be identified by estimating the PPA for each SNP within a predefined LD-independent segment. Genomic regions with PPA ≥ 0.8 for model 3 were considered pleiotropic; within such regions, the SNP with the highest PPA (≥ 0.5) was designated as the shared causal variant. The same predefined LD-independent blocks within European populations were used as testing regions for hyprcoloc, a Bayesian divisive clustering algorithm. hyprcoloc estimates the posterior probability of traits colocalizing within a given region, identifies the putative causal variant in this region, and the proportion of the posterior probability explained by that variant.

### 4.4. Multi-Trait Shared Loci Analysis

Moreover, a multi-trait analysis was conducted to identify shared signals using cofdr. Specifically, we explored shared genetic architecture between COVID-19 and multiple respiratory disorders simultaneously, focusing on traits that shared at least one validated locus with COVID-19 in pairwise comparisons. hyprcoloc was applied for further prioritization and validation of the results.

### 4.5. Shared Signals at the Gene Level—A Multi-Omics Approach

#### 4.5.1. Gene-Based Shared Signals Based on MAGMA (MAGMA-cofdr)

To identify genes shared between COVID-19 and other related medical conditions, we performed gene-based analysis using MAGMA (v1.09a) [84]. We first decorrelated SNP-based *p* values of involved traits to minimize the influence of sample overlap. We captured variants within the gene body only. Computed gene-based *p* values were converted into two-sided Z-values and input to cofdr. A posterior probability threshold of ≥0.9 was used to identify “shared” genes.

#### 4.5.2. Gene-Based Shared Signals from TWAS/spTWAS (TWAS/spTWAS-cofdr)

Second, we investigated shared genetically regulated effects on gene expression and alternative splicing across traits using transcriptome-wide association studies (TWASs) and splicing TWASs (spTWASs). We conducted a TWAS/spTWAS using decorrelated GWAS data to obtain gene-based Z-scores as input for cofdr. We employed MASHR-based eQTL/sQTL models [85] pre-trained on GTEx v8 data (49 tissues, N = 838) [86] (downloaded from PredictDB, https://predictdb.org/, accessed on 1 August 2023). Prediction models for each tissue were integrated with decorrelated GWAS statistics using S-PrediXcan (v0.7.3) [87], and association statistics across all tissues were combined using S-MultiXcan (v0.7.3) [88].

Similar to above, we applied a posterior probability (π_TT) threshold of 0.9. For splicing analysis, colocalized intron IDs discovered by spTWAS-cofdr were mapped to gene IDs using annotations from leafcutter (v.0.2.9) [89]. Meanwhile, for a gene containing multiple colocalized intron junctions and thus multiple π_TT values, we only report the largest one for that gene.

#### 4.5.3. Gene-Based Shared Signals from PWAS (PWAS-cofdr)

Similarly, we conducted a protein-wide association study (PWAS) to determine proteins that are jointly related to COVID-19 and other related respiratory disorders. In total, 1348 predictive models of plasma proteins fitted from 7213 European Americans (EAs) [90] were used for downstream PWAS using the tool FUSION (http://gusevlab.org/projects/fusion/, accessed on 1 January 2023) [91], based on harmonized decorrelated GWAS summary statistics. Significant shared proteins were defined as those with a posterior probability from cofdr greater than 0.8. This threshold is slightly more liberal than the 0.9 applied in MAGMA-cofdr, TWAS-cofdr, and spTWAS-cofdr analyses, reflecting the substantially smaller number of testable units in PWAS compared to gene-level analyses spanning approximately 18,000 genes across 49 tissues. With fewer testable units, a threshold of 0.8 retains adequate power to detect genuine shared signals while controlling false positives at a reasonable level, and is analogous to the PP.H4 >= 0.8 threshold widely adopted in colocalization analyses. Of note, in the context of local false discovery rates, Efron [79] suggested a threshold of 0.2, which corresponds to a posterior probability of association of 0.8.

To assess whether key findings are sensitive to the choice of posterior probability threshold, we repeated the identification of shared genes at thresholds of 0.80, 0.85, 0.90, and 0.95 across all omics layers and pairwise comparisons. Results are summarized in Supplementary Table S6.

The four gene-level approaches used here are not statistically independent, as MAGMA, TWAS, spTWAS, and PWAS all draw on the same GWAS summary statistics. They differ, however, in how genetic variants are linked to genes. MAGMA uses physical SNP-to-gene proximity, TWAS and spTWAS use eQTL and sQTL prediction models from GTEx v8, and PWAS uses plasma pQTL models. Each method therefore reflects a different biological layer of the same genetic variation. When a gene is identified by three or more methods, this represents convergent evidence across biological layers rather than independent statistical replication.

### 4.6. Differentiation Analysis at the SNP Level

#### 4.6.1. Pairwise Differentiation Analysis Using DDx

To identify genomic loci with divergent effects between COVID-19 and other respiratory traits, we performed a case–case GWAS using the “DDx” methodology we recently proposed (please see Ref. [92] for details of the method; custom pipeline available at https://github.com/xxxue96/ddx_cofdr_workflow, accessed on 1 March 2026). In essence, this approach treats one trait as the case and the other as the control and performs a GWAS. Sample overlap is also accounted for. SNPs with *p* < 5 × 10^−8^ in the derived case–case GWAS were considered significant for differential effects.

#### 4.6.2. Validation of Differentially Associated Variants Using CC-GWAS

CC-GWAS (v0.1.0) [93] was applied to validate the significant variants identified by DDx. Specifically, the algorithm computes case–case associations of two different disorders based on their respective case–control GWAS results. CC-GWAS combines two components, CC-GWASOLS and CC-GWASExact, to control type 1 error, and SNPs with OLS_*p*val < 5 × 10^−8^ and Exact_*p*val < 1 × 10^−4^ were considered statistically significant.

### 4.7. Multi-Trait Conditional Analysis Using mtCOJO

Whereas DDx performs a case–case comparison between any two traits, multi-trait conditional and joint analysis (mtCOJO, implemented in GCTA v1.94.1) [94] enables GWAS of a target phenotype Y conditioned on one or more genetically correlated phenotypes X. If X is genetically correlated with Y, SNP associations may become more or less significant after conditioning. mtCOJO can sometimes reveal genome-wide significant SNPs that are not significant in the unadjusted GWAS. Furthermore, mtCOJO allows for simultaneous conditioning on multiple traits.

We performed multivariate conditional analyses using mtCOJO to condition the effect of each SNP on COVID-19 upon genetically correlated respiratory traits. Genome-wide significant SNPs were identified in the conditional analysis at *p* < 5 × 10^−8^. MAGMA gene and gene-set analyses were conducted to identify genes and gene sets associated with the conditioned COVID-19 (MAGMA-mtCOJO). Tissue and cell-type specificity analyses were also performed using FUMA. Moreover, we applied TWAS, spTWAS, and PWAS to the conditioned GWAS to test the effects of disorder-specific variants on gene expression, splicing, and protein levels (TWAS-mtCOJO, spTWAS-mtCOJO, and PWAS-mtCOJO, respectively). Significant genes were identified at FDR < 0.01.

### 4.8. Differentiation Genetic Architecture at the Gene Level

Gene-level differentiation analysis was first performed by applying MAGMA to DDx-derived case–case GWAS (MAGMA-DDx), which involves physically mapping SNPs to genes. Next, to identify genes with divergent expression, splicing, or protein levels between traits, DDx-derived GWAS statistics were integrated with eQTL, sQTL, and pQTL data (TWAS-DDx, spTWAS-DDx, and PWAS-DDx, respectively). All differential genes were further compared with findings based on CC-GWASOLS case–case effect sizes. Due to the relatively large number of significant results, we mainly present findings with FDR < 0.01.

### 4.9. Post-GWAS Functional Annotation

Functional characterization and prioritization of the identified genomic risk loci were performed by FUMA (v1.6.1) [95]. We utilized the SNP2GENE module for functional annotation and GENE2FUNC for pathway enrichment analysis of colocalized genes. To identify tissues and cell types associated with differential effects, we conducted MAGMA gene-property analyses by integrating a case–case GWAS with GTEx v8 tissue expression data and 12 single-cell RNA-seq datasets.

### 4.10. Phenome-Wide Association (PheWAS) Analysis

The phenotypic associations of significant genes were investigated in the GWAS Atlas [96] to characterize their pleiotropic effects. The GWAS Atlas provides an integrated database of 4756 GWASs covering 3302 unique traits across 28 domains. Significant phenotypes classified into respiratory, cardiovascular, and immunological domains were extracted using Bonferroni-corrected *p*-values < 0.05/number of tested GWASs.

### 4.11. Multiple Testing and Error Control

Multiple testing correction was applied at each analytical layer using approaches appropriate to the statistical framework employed. At the SNP level, the cofdr algorithm operates within a posterior probability framework that directly controls the local false discovery rate. Shared variants were defined as those with a posterior probability of association (π_TT) ≥ 0.9. For colocalization validation using gwas-pw and hyprcoloc, a posterior probability of association threshold of 0.8 was applied, consistent with established practice in colocalization analysis. At the gene level, GWAS summary statistics for each trait were first processed within their respective original analyses (MAGMA, TWAS, spTWAS, PWAS), and the resulting gene-level statistics were then entered into cofdr. Shared genes were defined as those with π_TT ≥ 0.9 in cofdr analyses using MAGMA, TWAS, or spTWAS z-scores as input, and π_TT ≥ 0.8 for PWAS (see Section 4.5.3 for justification).

For differential gene-level analyses using MAGMA-DDx, TWAS-DDx, spTWAS-DDx, and PWAS-DDx, a significance threshold of FDR < 0.01 was applied. For pathway enrichment and tissue-property analyses using MAGMA gene-set analysis and FUMA, a more relaxed threshold of FDR < 0.05 was used. For differential SNP-level analyses using DDx, genome-wide significance was defined as *p* < 5 × 10^−8^. CC-GWAS validation additionally required an exact *p*-value < 1 × 10^−4^ to control type I error, as recommended by the original CC-GWAS methodology.

### 4.12. Guide to Supplementary Materials

The following Supplementary Tables are provided to facilitate interpretation of the results. Supplementary Table S1 contains GWAS dataset details and sample overlap estimates. Supplementary Table S2 reports pairwise shared loci and genes from cofdr, gwas-pw, hyprcoloc, MAGMA-cofdr, TWAS-cofdr, spTWAS-cofdr, and PWAS-cofdr analyses. Supplementary Table S3 reports multi-trait shared loci and genes. Supplementary Table S4 reports differential loci and genes from DDx/CC-GWAS, MAGMA-DDx, TWAS-DDx, spTWAS-DDx, and PWAS-DDx analyses; pathway enrichments (Table S4.7); tissue and cell-type enrichments (Tables S4.8 and S4.9); and drug enrichment results (Tables S4.10 and S4.11). Supplementary Table S5 reports mtCOJO conditional analysis results. Supplementary Table S6 presents a sensitivity analysis of key shared gene findings across posterior probability thresholds of 0.80, 0.85, 0.90, and 0.95. Supplementary Table S7 presents a cross-method concordance table showing which of the four omics methods (MAGMA, TWAS, spTWAS, PWAS) identified each top shared or differential gene.

## 5. Conclusions

In conclusion, we present a genome-wide, multi-omics and statistically rigorous framework that systematically studies shared and disease-discriminating genetic architecture between COVID-19 outcomes and multiple respiratory disorders. Our analyses highlight candidate COVID-discriminating pathways such as interferon signaling, alveolar epithelial/surfactant biology, and GM-CSF-linked mechanisms, providing novel hypothesis-generating insights for future functional and clinical investigations.

On the other hand, these genetic findings only represent one layer of a multi-factorial disease landscape. Epigenetic, microbiome, environmental and other factors may also make independent contributions to COVID-19 pathogenesis that future integrative studies will need to address.

We also prioritized COVID-specific candidate genes using conditional analyses (e.g., *FYCO1* and *HCN3*). In addition, we uncovered shared genetic signals between COVID-19 and a variety of other respiratory disorders. Together, these results shed light on the distinct and shared biological mechanisms underlying COVID-19 and other respiratory disorders, and provide an important resource of loci, genes, and pathways for follow-up functional studies, drug prioritization, and future polygenic risk prediction efforts.

## Figures and Tables

**Table 1:** Summary of analyzed GWASs.

Category	Trait	Abbreviation	Source	N_Cases	N_Controls	N_Effective
**COVID-19**						
	Severe COVID vs. population	A2	HGI R7	18,152	1,145,546	71,475
	Hospitalized COVID vs. population	B2	HGI R7	44,986	2,356,386	176,573
	Reported SARS-CoV-2 infection vs. population	C2	HGI R7	159,840	2,782,977	604,633
**Respiratory disorders**						
	Asthma	Asthma	GBMI	153,763	1,647,022	562,535
	Interstitial lung disease	ILD	PMID: 34594039	3313	644,534	13,184
	Idiopathic pulmonary fibrosis	IPF	GBMI	10,674	1,255,333	42,336
	Chronic obstructive pulmonary disease	COPD	GBMI	81,568	1,310,798	307,158
	All pneumonia ^a^	Pneu	Meta-analysis	58,600	688,122	—
	Bacterial pneumonia ^a^	Bact. pneu	Meta-analysis	22,399	682,697	—
	Viral pneumonia ^a^	Viral pneu	Meta-analysis	2373	667,718	—
	Influenza ^a^	Flu	Meta-analysis	4405	667,718	—

^a^ Meta-analyzed from PanUKBB and FinnGen using METAL (see Materials and Methods Section). Individual cohort details are provided in Supplementary Table S1. N_Effective not reported for meta-analyzed traits due to heterogeneity across component cohorts. HGI R7, COVID-19 Host Genetics Initiative Release 7 (PMID: 34237774); GBMI, Global Biobank Meta-analysis Initiative.

**Table 2:** Overview of shared and differential findings across all analyses.

Comparison	Shared Signals			Differential Signals		
	Loci (cofdr)	Validated Loci ^a^	Validated Genes ^b^	Loci (DDx)	Validated Loci ^c^	Validated Genes ^c^
**Severe COVID (A2)**						
A2—Asthma	32	4	23	36	13	22
A2—ILD	16	3	0	7	3	1
A2—IPF	**38**	**6**	**28**	20	9	11
A2—COPD	20	1	12	26	10	15
A2—Pneumonia ^d^	5	0	0	27	13	19
A2—Bact. Pneumonia ^d^	2	0	4	27	11	18
A2—Influenza ^d^	0	0	0	28	10	9
**Hospitalized COVID (B2)**						
B2—Asthma	31	3	20	**60**	**25**	**60**
B2—ILD	15	2	0	3	1	0
B2—IPF	**46**	**6**	**22**	17	10	8
B2—COPD	20	0	10	32	6	10
B2—Pneumonia ^d^	5	0	3	30	5	7
B2—Bact. Pneumonia ^d^	2	0	0	30	3	3
B2—Influenza ^d^	0	0	0	29	8	2
**Reported infection (C2)**						
C2—Asthma	10	2	16	**80**	**62**	**147**
C2—ILD	3	1	0	4	2	0
C2—IPF	10	2	2	19	12	8
C2—COPD	6	0	8	17	6	15
C2—Pneumonia ^d^	0	0	0	12	2	9
C2—Influenza ^d^	0	0	0	10	2	1
**Multi-trait colocalization**						
A2—Asthma/ILD/IPF/COPD	10	0	0	—	—	—
B2—Asthma/ILD/IPF ^e^	8	0	0	—	—	—
C2—Asthma/ILD/IPF ^e^	2	0	0	—	—	—
**Conditional (mtCOJO)**						
A2 conditioned	—	—	—	—	20 ^f^	4
B2 conditioned	—	—	—	—	5 ^f^	2
C2 conditioned	—	—	—	—	0 ^f^	2

^a^ Loci validated by cofdr + gwas-pw + HyPrColoc (triple-method validation). ^b^ Genes identified by ≥3 of four omics approaches (MAGMA, TWAS, spTWAS, PWAS). ^c^ Loci validated by DDx + CC-GWAS. ^d^ Meta-analyzed from PanUKBB and FinnGen. Viral pneumonia comparisons yielded no validated shared or differential results, reflecting the limited statistical power of the viral pneumonia meta-analysis (N_cases = 2373) rather than an absence of genetic overlap with COVID-19. ^e^ Using a relaxed cofdr posterior probability threshold of 0.8. ^f^ Number of SNPs that became more significant after conditioning on respiratory traits (disorder-specific SNPs). Bold values highlight comparisons with the largest numbers of validated findings.

**Table 3:** Validated shared loci between COVID-19 and respiratory disorders.

Comparison	Lead SNP	Chr:Pos	EA	β_COVID	β_Resp	P_COVID	P_Resp	Dir. ^a^	Nearest Gene(s)	PheWAS ^b^
A2–Asthma	rs34712979	4:106819053	A	−0.102	0.047	5.5 × 10^−10^	3.3 × 10^−18^	↑↓	*NPNT*	Resp
	rs34517439	1:78450517	A	0.11	0.034	1.0 × 10^−6^	3.6 × 10^−6^	↑↑	*DNAJB4*; *GIPC2*	Resp, Imm
A2–ILD	rs12802931	11:1236164	G	−0.103	0.583	2.4 × 10^−8^	6.8 × 10^−45^	↑↓	*MUC5B*	—
	rs2076295	6:7563232	G	0.068	0.155	1.5 × 10^−7^	7.7 × 10^−10^	↑↑	*DSP*	Resp
A2–IPF	rs12585036	13:113535741	T	0.138	−0.121	9.3 × 10^−19^	2.4 × 10^−11^	↑↓	*ATP11A*	Resp, Imm
	rs2277732	19:4723670	A	0.237	0.102	2.4 × 10^−56^	3.6 × 10^−9^	↑↑	*DPP9*	—
	rs12802931	11:1236164	G	−0.103	0.53	2.4 × 10^−8^	9.6 × 10^−112^	↑↓	*MUC5B*	—
	rs2076295	6:7563232	G	0.068	0.192	1.5 × 10^−7^	3.5 × 10^−40^	↑↑	*DSP*	Resp
B2–Asthma	rs34712979	4:106819053	A	−0.061	0.047	3.8 × 10^−8^	3.3 × 10^−18^	↑↓	*NPNT*	Resp
	rs13135092	4:103198082	G	0.085	0.067	2.7 × 10^−7^	5.6 × 10^−11^	↑↑	*SLC39A8*	Resp, Imm
B2–ILD	rs12802931	11:1236164	G	−0.067	0.583	9.9 × 10^−8^	6.8 × 10^−45^	↑↓	*MUC5B*	—
B2–IPF	rs75898026	13:113561082	A	0.086	−0.111	9.4 × 10^−14^	3.2 × 10^−9^	↑↓	*MCF2L*	Resp, Imm
	rs12802931	11:1236164	G	−0.067	0.53	9.9 × 10^−8^	9.6 × 10^−112^	↑↓	*MUC5B*	—
	rs35574495	19:4686988	T	0.125	0.104	8.7 × 10^−23^	1.8 × 10^−6^	↑↑	*DPP9*	—
C2–Asthma	rs13135092	4:103198082	G	0.046	0.067	2.5 × 10^−8^	5.6 × 10^−11^	↑↑	*SLC39A8*	Resp, Imm
C2–ILD	rs13243708	7:99616291	C	0.025	0.138	4.4 × 10^−7^	6.2 × 10^−8^	↑↑	*ZKSCAN1*	Imm
C2–IPF	rs732631	19:4719025	G	0.033	0.093	3.9 × 10^−12^	4.1 × 10^−9^	↑↑	*DPP9*	—

^a^ ↑↑, concordant direction (same allele increases risk for both traits); ↑↓, discordant direction (risk allele for one trait is protective for the other). ^b^ Lead SNP or proxy previously associated (Bonferroni-corrected *p* < 0.05) with respiratory (Resp) and/or immunological (Imm) phenotypes in GWAS Atlas. PheWAS—indicates no significant association in these domains. EA, effect allele. All loci validated by three independent methods (cofdr, gwas-pw, HyPrColoc). Genome-wide likelihood ratio tests confirmed significant shared genetic architecture for all comparisons shown (all LRT. *p* ≈ 0; see Supplementary Table S2.2).

**Table 4:** Key shared genes with multi-omics convergent evidence.

Gene	Comparison ^a^	MAGMA π_TT	TWAS π_TT (Tissue)	spTWAS π_TT (Tissue)	PWAS π_TT	Dir. ^b^	Functional Annotation
***COVID–IPF/ILD shared signals***							
*ATP11A*	A2–IPF	0.99	0.97 (Blood)	1.00 (Lung)	—	↑↓	Phospholipid flippase; lung fibrosis
*DPP9*	A2–IPF	1	—	0.99 (Lung)	—	↑↑	Dipeptidyl peptidase; known COVID–IPF locus
*TRIM4*	A2–IPF	0.96	—	0.99 (Lung)	—	↑↑	E3 ubiquitin ligase; IFN-β induction
*BAG6*	A2–IPF	0.99	—	0.98 (Lung)	—	↑↑	Co-chaperone; NK cell regulation
*ZNF3*	B2–IPF	1	0.93 (Blood)	1.00 (Lung)	—	↑↑	Zinc-finger transcription factor
*ZSCAN21*	B2–IPF	1	—	1.00 (Lung)	—	↑↑	Zinc-finger transcription factor
*FUT3*	C2–IPF	0.94	—	—	0.99	↑↑	Fucosyltransferase; glycan biology
***COVID–Asthma shared signals***							
*TCF19*	A2–Asthma	1	1.00 (Blood)	1.00 (Lung)	—	↑↓	Transcription factor; MHC region
*MED24*	A2–Asthma	0.96	0.96 (Blood)	1.00 (Lung)	—	↑↓	Mediator complex; transcriptional control
*INTS12*	A2–Asthma	0.99	0.99 (Blood)	1.00 (Lung)	—	↑↓	Integrator complex; snRNA processing
*NCOR1*	A2–Asthma	0.94	0.96 (Blood)	0.98 (Lung)	—	↑↓	Nuclear co-repressor; macrophage inflammation
*ZSWIM7*	B2–Asthma	0.99	0.97 (Lung)	0.99 (Lung)	—	↑↑	DNA repair; homologous recombination
*NPNT*	A2–Asthma	—	—	1.00 (Lung)	1	↑↓	Nephronectin; integrin signaling
*ICAM1*	B2–Asthma	0.96	0.98 (Blood)	—	—	↑↑	Cell adhesion; leukocyte migration
*BPTF*	A2–Asthma	0.93	—	0.93 (Lung)	—	↑↑	Chromatin remodeling (NURF complex)
*ACSL6*	B2–Asthma	0.99	0.91 (Blood)	—	—	↑↑	Long-chain fatty acid metabolism
*PIGL*	B2–Asthma	0.95	—	0.96 (Blood)	—	↑↑	GPI anchor biosynthesis
*GSDMA*	C2–Asthma ^c^	0.99	1.00 (Lung)	—	—	↑↓	Gasdermin A; pyroptosis
*MUC1*	C2–Asthma	1	—	1.00 (Lung)	—	↑↑	Mucin 1; mucosal barrier defense
*CCHCR1*	A2–Asthma	1	0.90 (Lung)	1.00 (Blood)	—	↑↓	HLA region; immune regulation
*PSMD3*	C2–Asthma	1	—	—	—	—	Proteasome subunit; protein degradation
*MICB*	A2–Asthma	0.91	—	—	—	—	MHC class I; NK cell activation
***Other notable shared signals***							
*SURF1*	C2–COPD	0.93	—	0.99 (Blood)	—	↑↑	Cytochrome c oxidase assembly
*DEPTOR*	B2–IPF	1	0.99 (Lung)	—	—	↑↑	mTOR inhibitor; autophagy regulation
*GSDMB*	B2–COPD	1	1.00 (Lung)	1.00 (Lung)	—	↑↓	Gasdermin B; airway inflammation

^a^ The comparison with the strongest evidence is shown. Many genes appear across multiple comparisons (see Supplementary Tables S2.3–S2.6 for complete results). For example, *GSDMA* was identified in A2–Asthma (0.95), B2–Asthma (0.99), A2–COPD (0.97), B2–COPD (0.98), C2–Asthma (0.99), and C2–COPD (0.99); *MED24* was identified across all three COVID phenotypes with both asthma and COPD. ^b^ ↑↑, concordant direction; ↑↓, discordant (opposing) direction. ^c^ π_TT is shown for C2–Asthma, which was also shared in five additional comparisons (see footnote a). Dashes (—) indicate that the gene was not identified at the specified omics layer at the threshold used (π_TT ≥ 0.9 for MAGMA/TWAS/spTWAS; ≥ 0.8 for PWAS). TWAS and spTWAS analyses were conducted across 49 tissues; for clarity, only results from lung and whole blood are shown here. Full tissue-specific results are available in Supplementary Tables S2.4 and S2.5.

**Table 5:** Key differential genes distinguishing COVID-19 from other respiratory disorders.

Gene	Comparison ^a^	Best DDx Z	Best DDx FDR	FDR_COVID	FDR_Resp	Omics ^b^	Dir. ^c^	Functional Theme
***Interferon signaling***								
*IFNAR2*	A2 vs. COPD	12.43	sT: 1.8 × 10^−31^	3.0 × 10^−36^	0.86	M, sT	COVID ↑	Type I IFN receptor
	A2 vs. IPF	8.37	sT: 6.7 × 10^−13^	3.0 × 10^−36^	0.92	M, sT	COVID ↑	
*OAS1*	A2 vs. IPF	6.51	sT: 4.0 × 10^−5^	6.8 × 10^−8^	0.74	M, sT	COVID ↑	2′–5′ Oligoadenylate synthetase
*OAS3*	A2 vs. IPF	4.41	T: 9.3 × 10^−3^	5.3 × 10^−5^	0.58	M, T	COVID ↑	2′–5′ Oligoadenylate synthetase
*TYK2*	A2 vs. IPF	−6.49	sT: 2.1 × 10^−7^	2.5 × 10^−8^	0.44	M, sT	COVID ↑	JAK–STAT signaling kinase
*IL10RB*	A2 vs. COPD	−5.55 ^d^	M: 1.6 × 10^−5^	1.4 × 10^−5^	0.41	M	COVID ↑	IL-10 receptor; anti-inflammatory
***Vascular/endothelial biology***								
*ARHGAP27*	A2 vs. Pneumonia	−6.67	M: 3.4 × 10^−7^	9.1 × 10^−7^	0.88	M, T, sT	Opposite	Rho GTPase; endothelial barrier
*RASIP1*	A2 vs. Pneumonia	−6.87	M: 1.9 × 10^−6^	5.9 × 10^−6^	0.61	M, T, sT	Opposite	Endothelial junction assembly
***Extracellular matrix/tissue remodeling***								
*THBS3*	A2 vs. COPD	−9.41	M: 1.6 × 10^−11^	1.1 × 10^−10^	0.16	M, sT	COVID ↑	Thrombospondin; ECM
*MTX1*	A2 vs. COPD	−7.59	sT: 7.7 × 10^−11^	7.9 × 10^−10^	0.3	M, sT	Opposite	Mitochondrial import; organellar stress
***Autophagy/intracellular trafficking***								
*FYCO1*	A2 vs. IPF	7.14	M: 7.3 × 10^−9^	3.7 × 10^−7^	0.82	M, sT	COVID ↑	Autophagosome transport
*FUT2*	A2 vs. IPF	6.47	M: 5.2 × 10^−5^	2.5 × 10^−7^	0.63	M, T	COVID ↑	Fucosyltransferase; mucosal immunity
*CALCOCO2*	B2 vs. Asthma	−4.96	T: 3.9 × 10^−4^	1.5 × 10^−2^	0.06	T	Opposite	Selective autophagy receptor (xenophagy)
*PLEKHM1*	A2 vs. Pneumonia	−6.79	M: 3.6 × 10^−7^	4.1 × 10^−6^	0.88	M, T, sT	Opposite	Autophagy–lysosomal trafficking
***NF-κB/inflammatory signaling***								
*NFKBIZ*	C2 vs. Pneumonia	7.42 ^d^	M: 5.4 × 10^−10^	3.0 × 10^−9^	0.27	M	COVID ↑	NF-κB inhibitor zeta; innate immunity
*FBRSL1*	A2 vs. Asthma	−4.91	M: 1.5 × 10^−6^	4.6 × 10^−6^	0.14	M, T, sT	COVID ↑	Fibrosin-like 1; chromatin regulation
*MAMSTR*	A2 vs. Pneumonia	−6.02 ^d^	M: 9.8 × 10^−7^	1.8 × 10^−5^	0.54	M	COVID ↑	MEF2-activating; transcriptional regulation
***Other notable differential signals***								
*DPP9*	A2 vs. Pneumonia	−6.89	M: 9.9 × 10^−10^	5.6 × 10^−10^	1	M, T, sT	COVID ↑	Dipeptidyl peptidase
*LTF*	A2 vs. Asthma	6.22 ^d^	M: 3.3 × 10^−7^	1.2 × 10^−6^	0.16	M	COVID ↑	Lactoferrin; innate immunity
*GSDMB*	B2 vs. Pneumonia	5.09	sT: 2.7 × 10^−4^	1.0 × 10^−3^	0.6	M, T, sT	Opposite	Gasdermin B; pyroptosis
*ABO*	C2 vs. Pneumonia	7.81	P: 7.3 × 10^−12^	2.5 × 10^−12^	0.9	M, T, P	COVID ↑	Blood group; coagulation

^a^ The comparison with the strongest or most illustrative result is shown; many genes are differential across multiple comparisons (see Supplementary Tables S4.3–S4.6 for complete results). ^b^ Omics layers in which the gene was identified as differential: M = MAGMA, T = TWAS, sT = spTWAS, P = PWAS. TWAS and spTWAS were conducted across 49 tissues; only lung and whole blood results are shown here. Full results are provided in Supplementary Tables S4.4 and S4.5. ^c^ “COVID ↑” = significant in COVID-19 only (FDR < 0.01) but not in the respiratory comparator (FDR > 0.05). “Opposite” = significant in COVID-19 but with opposing effect directions between COVID-19 and the comparator. ^d^ Best DDx Z is the Z-score from the omics method yielding the smallest DDx FDR for that gene and comparison. For MAGMA, Z-scores are derived from gene-level *p*-values with an arbitrarily assigned sign and do not carry directional meaning. For TWAS, spTWAS, and PWAS, the sign of Z indicates direction of effect in COVID-19 relative to the comparator. FDR_COVID and FDR_Resp refer to the original (non-differential) GWASs, demonstrating that these genes are driven primarily by COVID-19 association.

## Data Availability

The COVID-19 HGI summary statistics are publicly available via the Host Genetics Initiative portal (https://www.covid19hg.org/, accessed on 1 June 2024). The PanUKBB statistics can be accessed via the Pan-UK Biobank data repository (https://pan.ukbb.broadinstitute.org/, accessed on 1 June 2024). The FinnGen study results are downloadable through the FinnGen data portal (https://www.finngen.fi/, accessed on 1 June 2024). The Global Biobank Meta-analysis Initiative (GBMI) summary statistics are available via the GBMI data repository (https://www.globalbiobankmeta.org/, accessed on 1 June 2024). All intermediate multi-omics prediction models and supporting dataset tables generated during this study are preserved within the Supplementary Materials ZIP archive. The complete analytical framework, custom scripts, and pipeline implementations utilized to execute these analyses are openly accessible in our GitHub repository at https://github.com/xxxue96/ddx_cofdr_workflow (accessed on 1 March 2026).

## Supplementary Materials

The following supporting information can be downloaded at https://www.mdpi.com/article/10.3390/ijms27146536/s1.

## Abbreviations

The following abbreviations are used in this manuscript:

SNP	Single-nucleotide polymorphism
PPA/π_TT	Posterior probability of association
GWAS	Genome-wide association study
pQTL	Protein quantitative trait loci
PWAS	Protein-wide association study
sQTL	Splicing quantitative trait loci
spTWAS	Splicing transcriptome-wide association study
eQTL	Expression quantitative trait loci
TWAS	Transcriptome-wide association study
CC-GWAS	Case–case genome-wide association study
mtCOJO	Multi-trait conditional and joint analysis
FUMA	Functional mapping and annotation of genetic associations
LD	Linkage disequilibrium
LDSC	Linkage disequilibrium score regression
MAGMA	Multi-marker analysis of genomic annotation
FUSION	Functional analysis of summary expression data
PanUKBB	Pan-UK Biobank data resource
GBMI	Global Biobank Meta-analysis Initiative
GTEx	Genotype-Tissue Expression consortium
HGI	Host Genetics Initiative
COPD	Chronic obstructive pulmonary disease
ILD	Interstitial lung disease
IPF	Idiopathic pulmonary fibrosis
AEC	Alveolar epithelial cell
ECM	Extracellular matrix
DDx	Differential diagnosis
cofdr	A two-dimensional extension of the local true discovery rate (method for shared signal identification)
FDR	False discovery rate
LRT	Likelihood ratio test
MHC	Major histocompatibility complex
HLA	Human leukocyte antigen
PRS	Polygenic risk score
MAF	Minor allele frequency
